# Loss of IGF‐1R impairs DNA‐PKcs recruitment to chromatin leading to defective end‐joining

**DOI:** 10.1002/1878-0261.70266

**Published:** 2026-05-07

**Authors:** Matthew O. Ellis, Jack V. Mills, Wojciech Niedzwiedz, Valentine M. Macaulay

**Affiliations:** ^1^ UK‐DRI, Department of Clinical Neuroscience University of Cambridge Cambridge England; ^2^ Nuffield Department of Surgical Sciences University of Oxford Oxford England; ^3^ Institute of Cancer Research London England

**Keywords:** DNA repair, IGF‐1R, Irradiation, prostate cancer

## Abstract

The insulin‐like growth factor (IGF) axis regulates cancer cell proliferation, growth, invasion, and therapy resistance. Elevated expression of the type 1 IGF receptor (IGF‐1R) is linked to radioresistance and biochemical recurrence in prostate cancer, yet the molecular mechanisms underlying IGF‐1R–mediated DNA damage responses remain unclear. We investigated the role of IGF‐1R in DNA double‐strand break (DSB) repair by assessing chromatin recruitment of DNA repair proteins, repair pathway usage, and therapeutic sensitivity in cancer cell models with altered IGF‐1R status. Loss of IGF‐1R impaired DNA‐dependent protein kinase catalytic subunit (DNA‐PKcs) localisation to chromatin, resulting in defective non‐homologous end‐joining (NHEJ) and a compensatory reliance on alternative repair pathways, including microhomology‐mediated end‐joining (MMEJ). Modulating IGF‐1R expression restored radiosensitivity in poly (ADP‐ribose) polymerase (PARP) inhibitor–resistant breast cancer cells. IGF‐1R inhibition compromises canonical DSB repair and re‐sensitises resistant cancer cells to therapy, supporting its potential as a therapeutic strategy in homologous recombination–deficient tumours. Furthermore, IGF‐1R mutant cancers may benefit from targeted inhibition of the MMEJ pathway.

## Graphical abstract

IGF‐1R promotes radioresistance by facilitating DNA‐PKcs recruitment to chromatin, enabling non‐homologous end‐joining (NHEJ) repair of double‐strand breaks. Inhibition or loss of IGF‐1R disrupts this recruitment to damage sites, driving compensatory reliance on microhomology‐mediated end‐joining (MMEJ) repair. Our data support IGF‐1R inhibition as a therapeutic strategy in homologous recombination (HR)‐deficient tumours and implicate MMEJ inhibition in treatment of IGF‐1R mutant cancers.

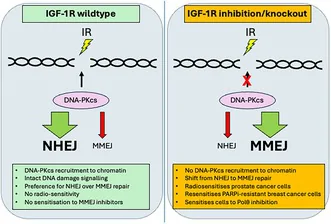

**Abbreviations**
4E‐BP1: eukaryotic translation initiation factor 4E‐binding protein 1
53BP1: p53‐binding protein 1
AKT: protein kinase B
AR: androgen receptor
ATM: ataxia telangiectasia mutated
BRCA1: breast cancer gene 1
cNHEJ: canonical non‐homologous end‐joining
CSK: cytoskeleton buffer
DDR: DNA damage response
DER: dose enhancement ratio
DNA‐PKcs: DNA‐dependent protein kinase catalytic subunit
DSB: double‐strand break repair
ELISA: enzyme‐linked immunosorbent assay
ERK: extracellular signal‐regulated kinase
FACS: fluorescence‐activated cell sorting
GATA2: GATA binding protein 2
gRNA: guide RNA
HR: homologous recombination
IF: immunofluorescence
IGF: insulin‐like growth factor
IGF‐1R: type 1 IGF receptor
INSR: insulin receptor
IP: immunoprecipitation
IR: ionising radiation
KAP1: KRAB‐associated protein 1
LC–MS/MS: liquid chromatography with tandem mass spectrometry
MEK: mitogen‐activated protein kinase
MMEJ: microhomology‐mediated end‐joining
mTOR: mechanistic target of rapamycin
NHEJ: non‐homologous end‐joining
NOM1: Nucleolar Protein With MIF4G Domain 1
NVB: Novobiocin
PARP: poly (ADP‐ribose) polymerase
PARPi: PARP‐1 inhibition
PCNA: proliferating cell nuclear antigen
PI3K: phosphatidylinositol 3‐kinase
PIKK: PI3K‐related kinase
POLθ: DNA polymerase theta
POLθi: POLθ inhibition
RAS: rat sarcoma virus
S6: ribosomal protein S6
TCEP: tris (2‐carboxyethyl) phosphine
TMB: tetramethylbenzidine

## Introduction

Men with prostate cancer may receive ionising radiation (IR) as primary treatment with curative intent, or to palliate symptoms of metastatic disease. Of those undergoing conventional fractionated IR for intermediate to high‐risk localised disease, reported 5–10 year treatment failure rates range from ~ 15% to 70% [1, 2, 3, 4, 5]. Attempts to characterise molecular mechanisms and gene signatures of radioresistance highlight the importance of the DNA damage response (DDR), cell cycle machinery, hypoxia, oxidative stress and epithelial–mesenchymal transition [6].

Our group and others identified signalling via the insulin‐like growth factor (IGF) axis as mediating resistance to a range of anti‐cancer treatments including endocrine therapy and IR [7]. Treatment resistance is mediated by activation of multiple signalling effectors including the phosphatidylinositol 3‐kinase (PI3K)‐protein kinase B (AKT)‐mechanistic target of rapamycin (mTOR) and rat sarcoma virus (RAS)‐mitogen‐activated protein kinase (MEK)‐extracellular signal‐regulated kinase (ERK) pathways following binding of IGFs to type 1 IGF receptors (IGF‐1Rs) [8] Previously, we reported that patients having radical IR experienced shorter recurrence‐free survival if their tumours contained high total IGF‐1R or cytoplasmic (internalised) IGF‐1R consistent with receptor activation [9]. Furthermore, cytoplasmic IGF‐1R was an independent predictor of post‐IR biochemical recurrence, including local recurrence within the radiation field indicating clinical radioresistance [9]. This association with cytoplasmic receptor may explain why *IGF1R* expression at the transcriptional level has not been identified in genetic signatures of prostate cancer radioresistance, although it was a component of a radioresistance signature in head and neck cancer [10, 11, 12].

The precise mechanism by which IGF‐1R modulates the DDR, particularly its role in DNA‐dependent protein kinase catalytic subunit (DNA‐PKcs) recruitment, remains poorly understood, despite evidence linking IGF‐1R to DSB repair. We and others reported that IGF‐1R inhibition or depletion enhances sensitivity to DNA‐damaging agents including IR and cytotoxic drugs in cancers of the prostate, breast, lung and brain [13, 14, 15, 16, 17, 18, 19, 20]. Furthermore, this effect appears to be independent of the ability of IGF‐mediated anti‐apoptotic activity, with data to support a role for IGF‐1R in regulating repair of double‐strand breaks (DSBs) by canonical non‐homologous end‐joining (cNHEJ) and homologous recombination (HR) [14, 21]. The aims here were to gain greater insight into the mechanism(s) through which IGF‐1R influences the DDR and explore novel approaches to exploit these dependencies.

Using *IGF1R* knockout (*IGF1R* ^*−/−*^) cells we show here that the IGF axis is responsible for the radio‐sensitisation observed previously upon knockdown or inhibition. Further, in IGF‐1 inhibited and *IGF1R* ^*−/−*^ cells, PARP‐1 inhibition (PARPi) or DNA polymerase theta (POLθ) inhibition (POLθi) increased sensitivity to radiation compared with IGF‐1R‐proficient cells. Lastly, in PARPi‐resistant SUM149 breast cancer cells, we demonstrate that IGF‐1 inhibition caused resensitisation to IR. These results highlight the potential for IGF axis inhibition in treatment of HR‐deficient tumours either in isolation or combination with PARPi or POLθi.

## Materials and methods

### Cell lines and treatments

Prostate cancer cell line DU145 (RRID:CVCL_0105) was cultivated and sourced from Cancer Research UK Laboratories (Clare Hall, Hertfordshire, UK) and 22Rv1 (RRID:CVCL_4Y35) from Professor Sir Walter Bodmer (Weatherall Institute of Molecular Medicine, University of Oxford, UK) purchased from ATCC. Professor Chris Lord (Institute of Cancer Research, London, UK) kindly provided breast cancer cell lines SUM149 (breast cancer gene 1 (*BRCA1*) mutant) and SUM149.B1.S* (‘SUM149 revertant’), a daughter clone harbouring an engineered secondary mutation that induced resistance to PARPi and restored damage‐induced RAD51 foci [22], obtained from ATCC – SUM149 (RRID:CVCL_3422). All cell lines used in this research have been authenticated within the past 3 years. Authentication was performed in accordance with current best practices and institutional guidelines to ensure the integrity and reproducibility of all experimental results. Briefly, cell line authentication was performed using short tandem repeat (STR) profiling consistent with ATCC standards. Genomic DNA was extracted and STR loci were amplified by PCR and cross‐referenced against the ATCC validated reference databases. Cell lines were tested regularly with MycoAlert (Lonza Rockland Inc., Basel, Switzerland) and were mycoplasma‐free. We used long R3‐IGF‐1 (Sigma Aldrich, St. Louis, MO, USA), non‐silencing Allstars siRNA and IGF‐1R siRNA (Hs_IGF1R_1; Qiagen, Hilden, Germany) as described previously [13, 14]. DNA‐PKcs inhibitor AZD7648 (AstraZeneca, Cambridge, UK), PARP‐1 inhibitor olaparib (Selleck Chem) and POLθ inhibitor novobiocin (NVB) (Selleck Chem, Houston, TX, USA) were dissolved in dimethyl sulfoxide at 10 mm (AZD7648), 20 mm (olaparib) or 50 mm (NVB). The latter was stored at 80 °C, the remainder at −20 °C. Xentuzumab (BI 836845, Boehringer) was kindly provided by Drs Ulrike Weyer‐Czernilofsky and Patrizia Sini (Boehringer Ingelheim, Ingelheim am Rhein, Germany) at 10 mg/mL, stored at 4 °C. Cells were irradiated in a Gamma‐Service Medical GmbH caesium‐137 irradiator (GSR D1).

### Knockout generation

Cells were transfected with plasmid pSpCas9(BB)‐2A‐GFP (PX458; Addgene #48138, Watertown, MA, USA) containing guide RNA (gRNA) sequences detailed in Table S1, using Lipofectamine 3000 (Thermo Fisher Scientific, Waltham, MA, USA) as per manufacturer's instructions. Single cells were sorted by fluorescence‐activated cell sorting (FACS) and the 20% most GFP‐positive cells were single‐cell sorted into multiple 96‐well plates. Clones were expanded and IGF‐1R expression assessed using IGF‐1R enzyme‐linked immunosorbent assay (ELISA), a modification of the phospho‐receptor ELISA used at Boehringer Ingelheim to assess IGF bioactivity [23, 24]. Cell monolayers at 70%–80% confluence were fixed (4% paraformaldehyde in Tris‐buffered saline (TBS) for 20 min), washed in 0.5% Triton X‐100 in TBS and quenched with 1.2% H_2_O_2_, 0.5% Triton X‐100 in TBS for 30 min. After further washing and blocking (5% bovine serum albumin (BSA) in 0.5% Triton X‐100 in TBS) for 1 h, blocking solution was aspirated and IGF‐1R antibody (Cell Signaling Technology Cat# 3027, RRID:AB_2122378, Danvers, MA, USA) diluted in blocking buffer was added and plates were incubated overnight at 4 °C. The following day cells were washed (0.5% Triton X‐100 in TBS), exposed to secondary antibody in blocking buffer and the plates incubated at room temperature for 1 h. After three washes in 0.5% Triton X‐100 in TBS and one with TBS, tetramethylbenzidine solution (TMB; Sigma Aldrich, T0440) was added to each well, incubated in the dark for 25 min and quenched by adding an equal volume of 2 m H_2_SO_4_. Absorbance (450 nm) was measured on a POLARstar OMEGA plate reader and expressed relative to mean WT value.

### Western blotting and chromatin fractionation

Cells were lysed in IGF‐1R lysis buffer described previously [14] or RIPA buffer (Sigma Aldrich) supplemented with phosphatase inhibitor cocktails 2 and 3 (Sigma Aldrich) and a protease inhibitor (Roche). Equal amounts of total protein were analysed by western blotting as described [25] using antibodies listed in Table S2.

Chromatin fractionation used a protocol described previously [26, 27]. Cells were incubated in cytoskeleton buffer (CSK; 100 mm NaCl, 300 mm sucrose, 3 mm MgCl_2_, 0.7% Triton X‐100, 10 mm PIPES, pH 7.0 with 0.3 mg/mL RNase A) for 3 min with shaking at 4 °C. After washing twice with ice cold phosphate buffered saline (PBS), a second incubation with CSK buffer and further PBS washes, chromatin was lysed in sodium dodecyl sulfate (SDS) lysis buffer (2% SDS, 10% glycerol, 25 mm Tris (2‐carboxyethyl) phosphine (TCEP), 62.5 mm Tris/HCl, pH 6.8) at room temperature (RT) and scraped into Eppendorf tubes. After brief vortexing, samples were boiled (95 °C, 10 min), centrifuged (15 493 xg, 1 min, RT), and protein concentrations of supernatants (chromatin) were assessed at OD_280_ (NanoDrop One, ThermoFisher). Cells were lysed and antibodies used for western blot are listed in antibody table (Table S2).

### Immunoprecipitation and mass spectrometric analysis of interactome

Cells were fractionated using NE‐PER™ Nuclear and Cytoplasmic Extraction Reagent kit (Thermofisher Scientific, 78 833) as per manufacturers' instructions. Nuclear extracts were used for IGF‐1R co‐immunoprecipitation (IP), using IGF‐1Rβ (CST 3027) antibody or IgG antibody as control (CST‐2729). IPs were collected with Protein A agarose beads and after the final wash, 5%–10% of the beads were removed and prepared for SDS/PAGE. The remaining beads, representing 90%–95% of the precipitate, were then washed with 20 mm HEPES for 5 min at 4 °C with end‐over‐end rotation, before collection by centrifugation at 3000× g, 3 min at 4 °C. Supernatant was discarded, and dry beads stored at −80 °C until use.

From the 5%–10% sample of beads retained for analysis, 50% were separated by SDS/PAGE alongside nuclear and whole cell extract input controls and analysed by western blot to check successful immunoprecipitation of IGF‐1R and fractionation of nuclear and cytoplasmic extracts by detection of fractionation markers Lamin A/C (nuclear marker) and β‐tubulin (cytoplasmic marker). The remaining 50% of retained beads were separated using SDS/PAGE and the gel was stained using Pierce™ Silver Stain for Mass Spectrometry (ThermoFisher Scientific, 24600) for detection of co‐immunoprecipitated proteins as per the manufacturer's protocol.

Subsequent steps were performed with the assistance of Dr Iolanda Vendrell (Mass Spectrometry Scientist, MRC Oxford Institute for Radiation Oncology, University of Oxford). Samples containing 90%–95% of each precipitate, which had been stored at −80 °C, were defrosted, resuspended in 50 μL of 2 m urea in 20 mm HEPES pH 8 and 1.3 μL of DTT (200 mm, final concentration of 5 mm) and incubated for 30 min at RT. After incubation, indole‐3‐acetic acid (20 mm) (Sigma Aldrich, I1149) was added and samples were incubated for a further 30 min at RT, shielded from light. To reduce the concentration of urea to < 2 m to allow trypsin to function correctly, HEPES pH 8 (20 mm) was added to each sample, along with trypsin (0.2 μg/μl) (V5111; Promega, Madison, WI, USA). Samples were incubated at 37 °C overnight in a ThermoMixer with agitation (900 rpm). After halting trypsin digestion with 1% trifluoroacetic acid (TFA) (Sigma Aldrich, T6508), samples were gently vortexed and centrifuged at 2000 x g for 5 min at 4 °C. Supernatants (tryptic digests) were then transferred to fresh Eppendorf tubes and desalted using SOLA‐HRP cartridges (60409; Thermo Fisher Scientific) as per manufacturers' instructions. Eluted peptide samples were then dried in a Savant™ SpeedVac™ (SPD120; Thermo Fisher Scientific) and stored at −20 °C until use.

Liquid Chromatography tandem mass spectrometry (LC–MS/MS) analysis was performed using an Ultimate 3000‐Ultra High Performance Liquid Chromatography (UHPLC) system connected to an Orbitrap Fusion‐Lumos Tribid Mass Spectrometer (ThermoFisher), located at the Proteomics Facility, Target Discovery Institute, Nuffield Department of Medicine Research Building, Oxford. Initial analysis used Mascot protein identification software (http://www.matrixscience.com/) and the Swiss‐Prot protein database (https://www.uniprot.org/) without defining a species or database for the peptides present. The PEAKS proteomics software (https://www.bioinfor.com/peaks‐studio/) was used to analyse the data against the Swiss‐Prot Human protein.

### Clonogenic assay

Cells were transfected with control or IGF1R siRNAs as described [13]. After 48 h, cultures were reseeded as single cells into 10 cm dishes and the following day irradiated at 2–8 Gy in a Caesium‐137 source (IBL 637 irradiator, CIS Bio International, France). Dishes were incubated at 37 °C in 5% CO_2_ for 10–14 days. When colonies of ~ 50 cells were visible, colonies were stained and counted on a GELCount automated colony counter (Oxford Optronix).

### Immunofluorescence

Cells were fixed in 4% paraformaldehyde in PBS, permeabilised in 0.2% Triton X‐100 in PBS and blocked in 10% FBS in PBS. After overnight incubation at 4 °C with primary antibodies (Table S2) in 0.1% FBS in PBS, secondary antibodies were added in 0.1% FBS in PBS for 1 h at room temperature. Images were acquired using the Opera Phenix Spinning Disk Confocal microscope and analysed using Harmony High‐Content Imaging and Analysis software (Perkin Elmer, Waltham, MA, USA).

### Cell‐based assays

Proliferation rate was monitored by plating cells in 96‐well plates in duplicate (10^3^ cells/well) and confluence monitored using the Incucyte Live‐Cell Analysis System (Sartorius). Cell viability assays used CellTiter Glo (Promega) according to the manufacturer's protocol. Transfections with siRNA were performed 48 h before re‐seeding for clonogenic assay. Treatments and solvent controls were added 4 h before irradiation, and clonogenic assays were performed as described previously [13]. Cell‐based ELISAs used a modification of the method described previously [23] to assay total and phospho‐Y1135/1136 IGF‐1R using primary antibodies #3027 (Cell Signaling Technology Cat# 3027, RRID:AB_2122378) and #3024 respectively (Cell Signaling Technology Cat# 3024, RRID:AB_331253).

### MMEJ reporter assay

Microhomology‐mediated end‐joining (MMEJ) assay was performed as described [28]. Briefly, plasmid pDVG94 was linearised, transfected into 22Rv1 cells, and plasmid DNA isolated 48 h later. The region around the break site was amplified by polymerase chain reaction (PCR) and digested with BstXI. If the plasmid was repaired by cNHEJ, no BstXI restriction site will be present in the 180 bp product. Conversely, MMEJ creates a BstXI restriction site, cleaving the 180 bp PCR product into 120 and 60 bp products. And digestion and gel electrophoresis of the PCR product was used to indicate the relative amounts of cNHEJ vs MMEJ repair. Plasmid pDVG94 was digested with EcoRV and AfeI (New England Biolabs) and the 7739 bp fragment gel purified. As controls for compromised cNHEJ, cells were treated with 1 μm AZD7648 (or solvent control) for 4 h, followed by transfection with 7739 bp linearised pDVG94 fragment. After 48 h, plasmid DNA was isolated, amplified by qPCR using primers DAR5 and FM30 (Table S1), and digested with BstXI. Products were separated by agarose gel electrophoresis to visualise the intact 180 bp (black arrow) and 120 bp (red arrow) products [28]. These were visualised by ethidium bromide staining. Product intensities were quantified using ImageJ software (RRID:SCR_003070) and data were analysed by one‐way ANOVA to determine the statistical significance of differences in relative MMEJ.

### Statistical analysis

Statistical significance was determined using GraphPad Prism version 8.0.1 for Mac (GraphPad Software, San Diego, CA, USA) (RRID:SCR_002798). One‐way ANOVA was used to assess differences between the means of more than two independent unrelated groups, and two‐way ANOVA was used for trends between relative band intensities on western blots and clonogenic survival curves. P‐values for significance were calculated and stated in corresponding figure legends.

## Results

### IGF1R gene silencing induces radiosensitivity in human prostate cancer cells

We previously showed that IGF‐1R knockdown or inhibition enhanced radiosensitivity of DU145 prostate cancer and glioblastoma cells [13, 14, 20]. Here, we confirmed the radiosensitivity phenotype in IGF‐1R depleted DU145 cells, with similar radio‐sensitisation upon IGF‐1R depletion in 22Rv1 cells (Fig. 1A, Fig. S1A,B). In both cell lines, IGF‐1R depletion resulted in dose enhancement ratios (DERs) of at least 3.0 at 4 Gy and above (Table S3), values of potential clinical relevance [29]. This enhancement suggests that IGF‐1R loss exacerbates DNA repair deficiencies, increasing cell death upon radiation exposure.

**Fig. 1:**
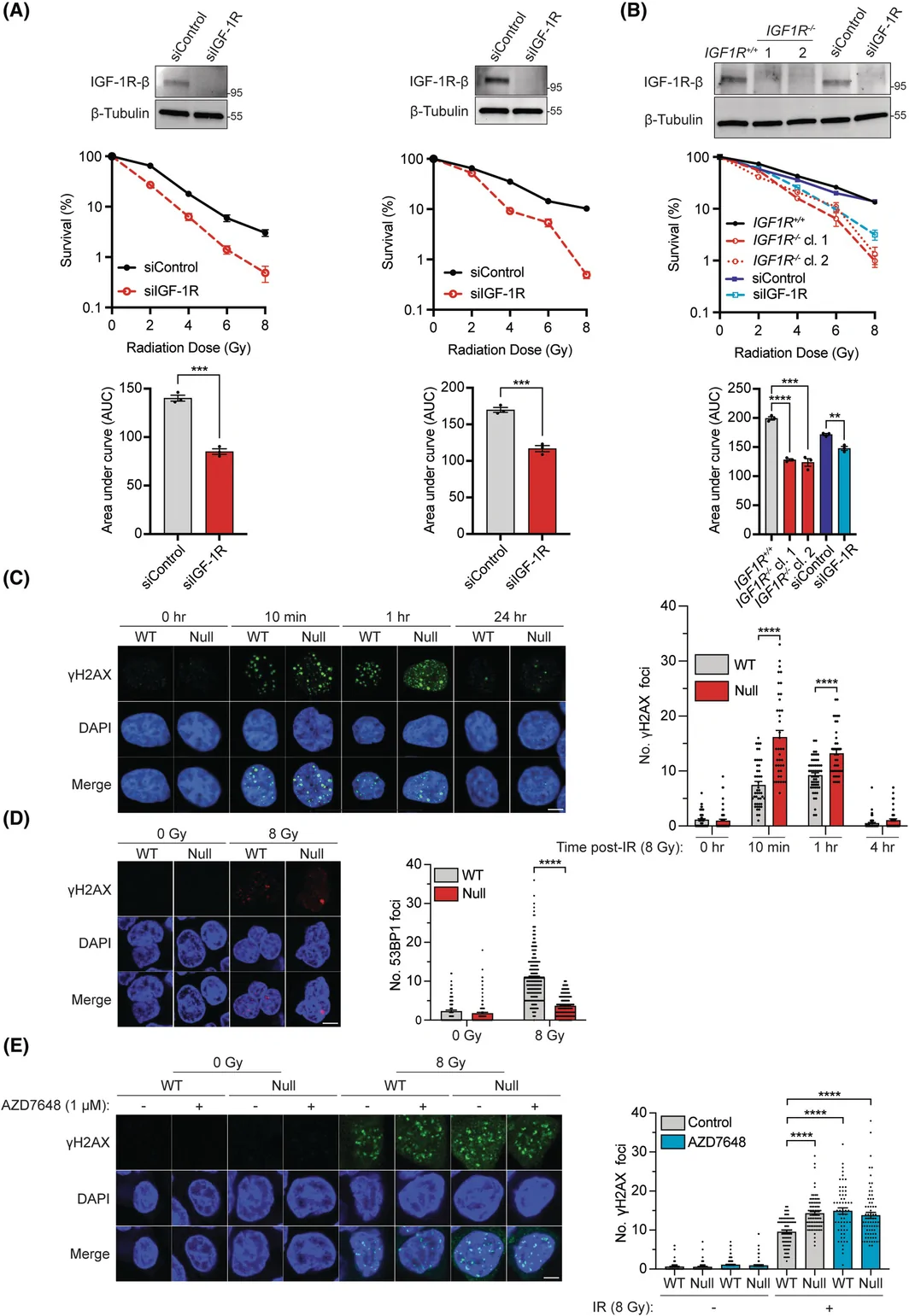
IGF‐1R depletion enhances prostate cancer cell radiosensitivity with evidence of non‐homologous end‐joining impairment. (A) Prostate cancer cell lines DU145 and 22Rv1 were transfected with control (siControl) or type 1 insulin‐like growth factor receptor (IGF‐1R) (siIGF‐1R) siRNAs. After 48 h cultures were reseeded as single cells into 10 cm dishes and the following day irradiated at indicated doses and whole cell lysates were collected to measure IGF‐1R depletion. After 10–13 days, colonies were stained and counted using the GelCount. Graphs: cell survival as % unirradiated controls (one representative repeat of 3 independent biological repeats, error bars represent ±SEM) in DU145 (left) and 22Rv1 (right). Inset: Western blots of parallel siRNA‐transfected cultures confirming IGF‐1R depletion. Area under curve: Unpaired t‐test: ***P < 0.001. Representative clonogenic assay dishes in Figs 1A,B. (B) Radiosensitivity assay comparing effects of IGF‐1R depletion or deletion in 22Rv1 cells. Parental cells were transfected with siControl or siIGF‐1R and seeded after 48 h as well as one IGF1R+/+ clone and two IGF1R−/− clones. Cells were irradiated the following day at indicated doses and stained and counted after 10–13 days using the GelCount. Graph: cell survival as % unirradiated controls (one representative repeat of 3 independent biological repeats, error bars represent ±SEM) Area under curve: Unpaired t‐test: **P < 0.01, ***P < 0.001, ****P < 0.0001. (n = 3 independent experiments, error bars represent ±SEM). Representative clonogenic assay dishes in Fig. 2F. (C) Immunofluorescence staining of one IGF1R+/+ (wild‐type (WT)) and one IGF1R−/− (null) clone after fixation at indicated time intervals after 8 Gy irradiation (IR). Cells were stained for γH2AX (green) and DAPI (blue) and foci were plotted (n = 3 independent experiments, error bars represent ±SEM). 10 min: ****P < 0.0001, 1 h: ****P < 0.0001 by two‐way ANOVA. Scale bar = 10 μM. (D) Immunofluorescence staining of one IGF1R+/+ (WT) and one IGF1R−/− (Null) clone after fixation pre‐ and 10 min post‐IR (8 Gy). Cells were stained for p53‐binding protein 1 (53BP1) (red) and DAPI (blue) and foci were plotted (n = 3 independent experiments, error bars represent ±SEM). 8 Gy: ****P < 0.0001 by two‐way ANOVA. Scale bar = 20 μM. (E) Immunofluorescence staining of one IGF1R+/+ (WT) and one IGF1R−/− (Null) clone after fixation pre‐ and 10 min post‐IR. Indicated cells were treated with DNAPKi (AZD7648) (1 μm) for 4 h prior to IR (8 Gy). Cells were stained for γH2AX (green) and DAPI (blue), foci were plotted (n = 3 independent experiments, error bars represent ±SEM). 8 Gy: IGF1R+/+ ‐AZD7648 vs + AZD7648 (****P < 0.0001), IGF1R+/+ ‐AZD7648 vs IGF1R−/− ‐AZD7648 (****P < 0.0001), IGF1R+/+ ‐AZD7648 vs IGF1R−/− + AZD7648 (****P < 0.0001) by one‐way ANOVA. Scale bar = 10 μM. Imaging was performed using the Opera Phenix Spinning Disk Confocal microscope and foci were analysed using Harmony High‐Content Imaging and Analysis software (Perkin Elmer).

We selected 22Rv1 in preference to DU145 as the host to generate a stable model of compromised IGF‐1R function. This is because 22Rv1 cells express wild‐type (WT) TP53 and are androgen receptor (AR) positive, allowing cross‐talk between the IGF‐ and AR‐ axes, as occurs in clinical prostate cancers [30, 31]. We created 22Rv1 *IGF1R* ^*−/−*^ clones using CRISPR/Cas9 technology to target exon 2 of the *IGF1R* gene and in parallel generated WT *IGF1R* ^*+/+*^ clones by serial dilution from bulk 22Rv1 cultures. Candidate *IGF1R* ^*−/−*^ clones were screened in a cell‐based IGF‐1R enzyme‐linked immunosorbent assay (ELISA) (Fig. S1C) followed by western blotting, confirming IGF‐1R loss in most screen‐selected clones. All signalling readouts were under basal (unstimulated) conditions; accordingly, we observed only a minor reduction in phosphorylation of ribosomal protein S6 (S6) (pS6) and an apparent increase in phospho‐eukaryotic translation initiation factor 4E‐binding protein 1 (p4E‐BP1) in *IGF1R* ^*−/−*^ clones (Fig. S1D,E), perhaps reflecting cross‐talk within the IGF‐1R‐AKT–mTOR axis [32]. Sanger sequencing confirmed disruption of *IGF1R* exon 2 sequence in two *IGF1R* ^*−/−*^ clones taken forward for further study (Fig. S1F).

We also tested cellular response to ligands, observing IGF‐induced increase in receptor phosphorylation in *IGF1R* ^*+/+*^ cells, but not in *IGF1R* ^*−/−*^ cells, whereas insulin induced similar receptor phosphorylation in both backgrounds (Fig. S2A,B). These results are consistent with loss of IGF‐1R and conserved INSR function. In proliferation and clonogenic assays, *IGF1R* ^*−/−*^ clones exhibited similar growth and survival to *IGF1R* ^*+/+*^ cells (Fig. S2C,D), consistent with reports that IGF‐1R is relatively unimportant for 2D growth [33]. However, the critical role of IGF‐1R in the context of DNA damage repair became apparent in radiation survival assays. Both acute siRNA‐mediated IGF‐1R depletion and chronic IGF‐1R deletion enhanced radiosensitivity, producing similar effects in *IGF1R* ^*+/+*^ cells and siControl transfectants, and comparable dose‐dependent sensitisation of *IGF‐1R* ^*−/−*^ clones and IGF‐1R depleted cells when plated in parallel (Fig. 1B, Fig. S2E). Overall, the equivalence of phenotypes suggested minimal long‐term compensation. Both IGF‐1R loss and depletion generated DERs of ≥2.0 at 4 Gy IR (Table S3). Even this relatively low DER value may be useful given that it applies at the clinically relevant single fraction dose of 4 Gy [29].

To explore how IGF‐1R depletion influences the DDR, we analysed the formation and resolution of IR‐induced damage foci. Although γH2AX foci form at a variety of DNA lesions, the majority generated post‐IR are at DSBs [34]. *IGF1R* ^*+/+*^ and *IGF1R* ^*−/−*^ clones were fixed at intervals after 8 Gy IR, the dose that caused the greatest difference in radiosensitivity between *IGF1R* ^*+/+*^ and *IGF1R* ^*−/−*^ cells (Fig. 1B), and γH2AX foci were visualised by immunofluorescence (IF). Time intervals (10 min to 24 h) were chosen based on our prior data in DU145 cells [14] and reported radiation responses in 22Rv1 cells [35]. Unirradiated cells contained few γH2AX foci, with an increase post‐IR as expected. There were significantly more foci in *IGF1R* ^*−/−*^ cells at 10 min and 1 h post‐IR, but at 24 h γH2AX foci had largely resolved in both *IGF1R* ^*+/+*^ and *IGF1R* ^*−/−*^ cells (Fig. 1C). The speed of γH2AX focus resolution serves as an indicator of the major repair pathway(s) involved. IR‐induced DSBs are repaired with two‐component kinetics: cNHEJ accomplishes both the rapid and slow components in G0‐G1 cells, rejoining ~ 80% breaks within 4 h, and is also responsible for rapid DSB repair in S and G2, while HR is utilised for slow repair in G2 cells [36, 37]. Here, *IGF1R* ^*−/−*^ 22Rv1 cells showed a DSB repair defect within 1 h of IR, suggesting a cNHEJ defect. We previously observed a similar early defect in IGF‐1R inhibited DU145 cells, in that model unrepaired breaks persisted at 24 h with reporter evidence of defects in both cNHEJ and HR, more pronounced for cNHEJ [14].

We employed two strategies to probe effects of IGF‐1R loss on cNHEJ in isogenic 22Rv1 cells. First, we assessed p53‐binding protein 1 (53BP1) foci. The p53‐binding protein 53BP1 inhibits end‐resection, a key step in the initiation of HR, and negatively regulates resection nucleases, thus promoting cNHEJ and serving as a marker of cNHEJ function [38, 39]. Imaging cells before and 10 min post‐IR, we detected significantly more 53BP1 foci in *IGF1R* ^*+/+*^ cells than in *IGF1R* ^*−/−*^ cells (Fig. 1D). The same pattern was apparent when we repeated the assessment in two independent *IGF1R* ^*+/+*^ and two *IGF1R* ^*−/−*^ clones (Fig. S2F). Next, we tested the impact of DNA‐PKcs inhibition on γH2AX focus formation. *IGF1R* ^*+/+*^ and *IGF1R* ^*−/−*^ cells were treated for 4 h with solvent (control) or AZD7648, an inhibitor of the catalytic subunit of DNA‐dependent protein kinase (DNA‐PKcs), then irradiated (8 Gy) and fixed after 10 min. We observed an IR‐induced increase in γH2AX foci in both *IGF1R* ^*+/+*^ and *IGF1R* ^*−/−*^ cells, with a further increase in AZD7648‐treated *IGF1R* ^*+/+*^ cells. Control‐treated *IGF1R−/−* cells contained significantly more γH2AX foci than control‐treated *IGF1R* ^*+/+*^ cells, consistent with the previous result (Fig. 1C) but focus numbers did not increase in *IGF1R* ^*−/−*^ cells upon AZD7648 treatment (Fig. 1E). Thus DNA‐PKcs inhibition did not further increase γH2AX foci in cells lacking IGF‐1R. This implies that with respect to post‐IR DSB repair, IGF‐1R operates in the same pathway as DNA‐PKcs. These results are consistent with an epistatic relationship between IGF‐1R and NHEJ in 22Rv1 prostate cancer cells, and IGF‐1R loss primarily impairing DSB repair by cNHEJ [14].

### IGF‐1R null cells exhibit defective DNA‐PKcs autophosphorylation

We then investigated the functional basis linking the IGF axis to DNA repair pathways. Having observed that DNA‐PKcs inhibition impaired the DDR in *IGF1R* ^*+/+*^ but not *IGF1R* ^*−/−*^ cells (Fig. 1E), we speculated that deleting IGF‐1R might itself compromise DNA‐PKcs function. Western blots on whole cell lysates collected 10 min post‐IR confirmed this, showing a clear reduction in IR‐induced S2056 DNA‐PKcs autophosphorylation in *IGF1R* ^*−/−*^ cells compared to *IGF1R* ^*+/+*^ cells (Fig. 2A). The disparity was apparent at all IR doses and was most marked at 8 Gy, the highest irradiation dose we tested. In contrast, phosphorylation of the ataxia telangiectasia mutated (ATM)‐substrate KRAB‐associated protein 1 (KAP1) at serine 824 (S824) was not impaired by IGF‐1R loss (*IGF1R* ^*−/−*^ cells, Fig. 2A).

**Fig. 2:**
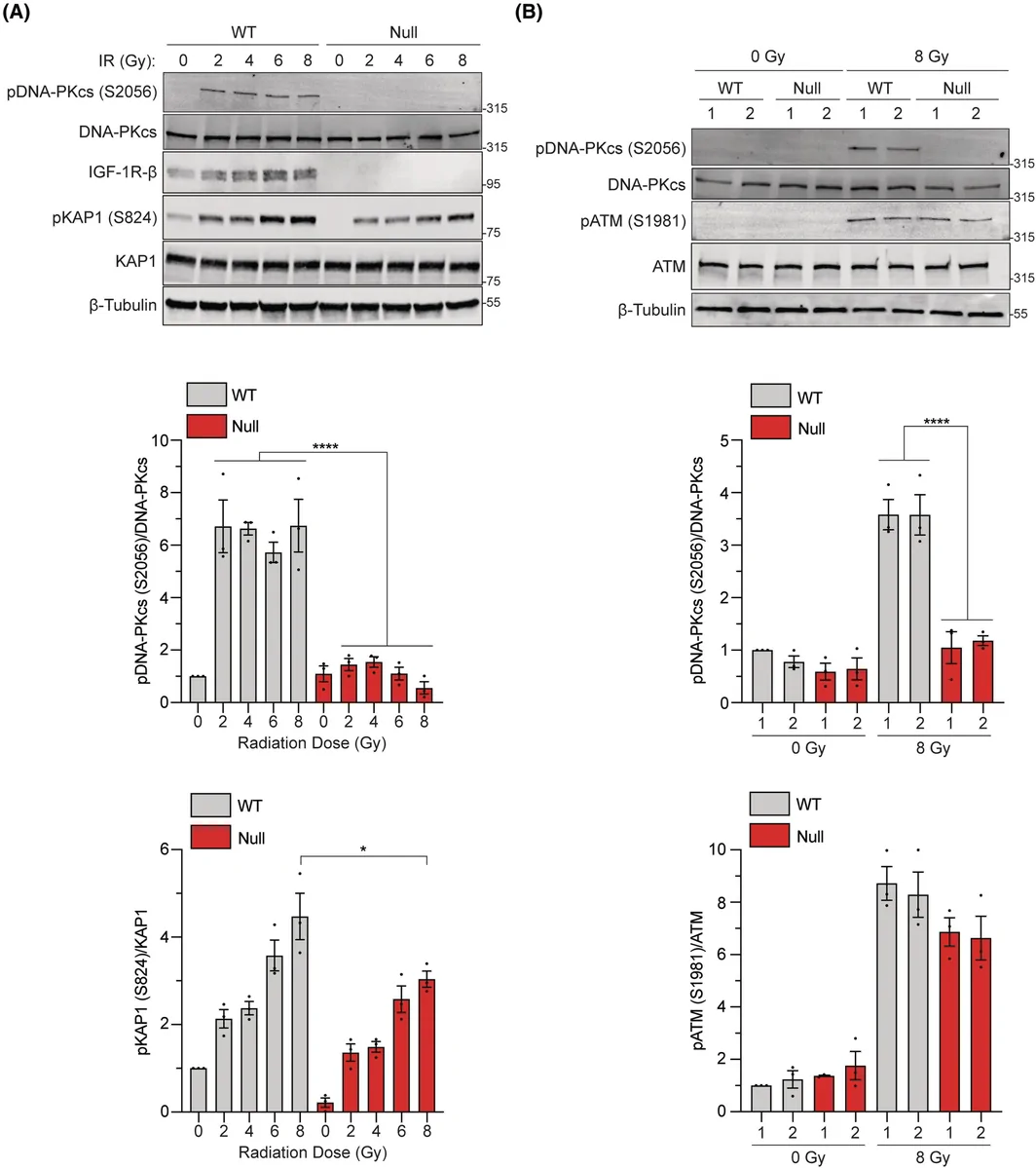
Loss of IGF‐1R reduces DNA‐PKcs phosphorylation after irradiation. (A) Whole cell lysates were obtained pre‐ and 10 min post‐irradiation (IR) at indicated doses from one type 1 insulin‐like growth factor receptor (IGF1R)+/+ (wild‐type (WT)) and one IGF1R−/− (null) clone. Above: Western blot representative of three independent repeats. Below: Quantification of bands in three independent repeats (error bars represent ±SEM). IGF1R+/+ vs IGF1R−/− (*P < 0.05, ****P < 0.0001) by one‐way ANOVA. (B) Whole cell lysates were obtained pre‐ and 10 min post‐IR (8 Gy) from two IGF1R+/+ and two IGF1R−/− clones. Above: Western blot representative of three independent repeats. Below: Quantification of bands in three independent repeats (error bars represent ±SEM). Phospho‐DNA‐dependent protein kinase catalytic subunit (DNA‐PKcs) (serine 2056) 8 Gy: IGF1R+/+ clone 1 vs IGF1R−/− clone 1 (****P < 0.0001), IGF1R+/+ clone 1 vs IGF1R−/− clone 2 (****P < 0.0001), IGF1R+/+ clone 2 vs IGF1R−/− clone 1 (****P < 0.0001), IGF1R+/+ clone 2 vs IGF1R−/− clone 2 (****P < 0.0001) by one‐way ANOVA. All western blot analyses were performed using ImageJ software where all band intensities were relative to the average of IGF1R+/+ intensities at 0 Gy.

To exclude the clone‐specific effect, we repeated the experiment using two *IGF1R* ^*+/+*^ and two *IGF1R* ^*−/−*^ clones (Fig. 2B). Given the conserved nature of the PI3K‐related kinase (PIKK) domains of DDR kinases [40], we compared IR‐induced phosphorylation of ATM (S1981) and DNA‐PKcs (S2056) 10 min after 8 Gy (Fig. 2B). Both *IGF1R* ^*+/+*^ clones phosphorylated ATM and DNA‐PKcs in response to IR, but there was no increase in pS2056 DNA‐PKcs post‐IR in either *IGF1R* ^*−/−*^ clone. Quantification of three independent replicates confirmed this (Fig. 2B). In contrast, there was no significant difference in ATM phosphorylation in IGF‐1R proficient or deficient cells (Fig. 2B), consistent with unimpaired phosphorylation of KAP1 (Fig. 2A), a known ATM substrate [41, 42]. The striking lack of DNA‐PKcs phosphorylation in IGF‐1R‐deficient cells is a novel finding that implicates IGF‐1R, either directly or indirectly, in modulation of DNA damage‐induced DNA‐PKcs autophosphorylation.

We considered whether altered signalling could explain this effect. Although AKT has been linked to DNA‐PKcs activation and HR suppression [43, 44, 45, 46, 47, 48], we observed no consistent difference in downstream mTOR readouts (pS6 and p4E‐BP1) in *IGF1R* ^*+/+*^ vs *IGF1R* ^*−/−*^ cells (Fig. S1D,E). There is also evidence of key DDR proteins controlling AKT activity [49, 50, 51, 52], suggesting a complex network of interactions in response to damage, of which data presented here indicate IGF‐1R is a contributor.

Since DNA‐PKcs is primarily nuclear [53], and we and others have reported that activated IGF‐1R can undergo nuclear translocation [54, 55, 56], we asked whether the two proteins interact. We immunoprecipitated the IGF‐1Rβ‐subunit from nuclear extracts of the two *IGF1R* ^*+/+*^ and two *IGF1R* ^*−/−*^ clones in which we had compared IR‐induced DNA‐PKcs phosphorylation (Figs 2A,B), and analysed by liquid chromatography with tandem mass spectrometry (LC–MS/MS) under non‐irradiated conditions. The protein dataset included previously identified interactors of nuclear IGF‐1R including proliferating cell nuclear antigen (PCNA), importin‐β, Ran‐BP2, Nucleolar Protein With MIF4G Domain 1 (NOM1) and GATA binding protein 2 (GATA2) [56, 57, 58, 59]. Correlating with data from the Larsson group, we found that DNA‐PKcs was identified as the top ranked hit detected significantly more frequently in *IGF1R* ^*+/+*^ vs *IGF1R* ^*−/−*^ cells (Table S4). The nuclear IGF‐1R‐DNA‐PKcs association was further validated by co‐IP/immunoblotting, in untreated and IR‐treated cells, confirming DNA‐PKcs co‐precipitation with IGF‐1R (Fig. S3A). This supports a functional nuclear IGF‐1R‐DNA‐PKcs interaction, providing a mechanistic link to the observed cNHEJ defect.

### Loss of functional IGF‐1R impairs recruitment of repair proteins to chromatin

DNA‐PKcs autophosphorylation occurs upon DNA‐PKcs recruitment to DNA ends [60, 61]. Given the impaired pS2056‐DNA‐PKcs signal and reduced cNHEJ activity in *IGF1R* ^*−/−*^ cells, we hypothesised that IGF‐1R loss may disrupt the recruitment of cNHEJ factors to chromatin. Standard IF approaches are limited by small foci and RNA‐dependent chromatin association [26]. To overcome this, we used a detergent‐ and RNase‐based extraction method to assess chromatin‐bound repair proteins via western blot [26, 27, 62]. Chromatin fractionation from *IGF1R* ^*+/+*^ and *IGF1R* ^*−/−*^ clones was collected alongside corresponding whole cell extracts at the indicated time points post‐IR.

Western blots revealed that both DNA‐PKcs and Ku80 were recruited to chromatin at significantly higher levels in *IGF1R* ^*+/+*^ cells 10 min after IR compared to *IGF1R* ^*−/−*^ cells, and this trend continued at 1 h and 4 h post‐IR (Fig. 3A), indicative of normal DNA repair in cells with intact IGF‐1R signalling [26, 27, 62, 63]. In *IGF1R* ^*+/+*^ cells, IGF‐1R was not recruited to chromatin, suggesting that IGF‐1R does not directly load DNA‐PKcs onto DNA (Fig. 3A).

**Fig. 3:**
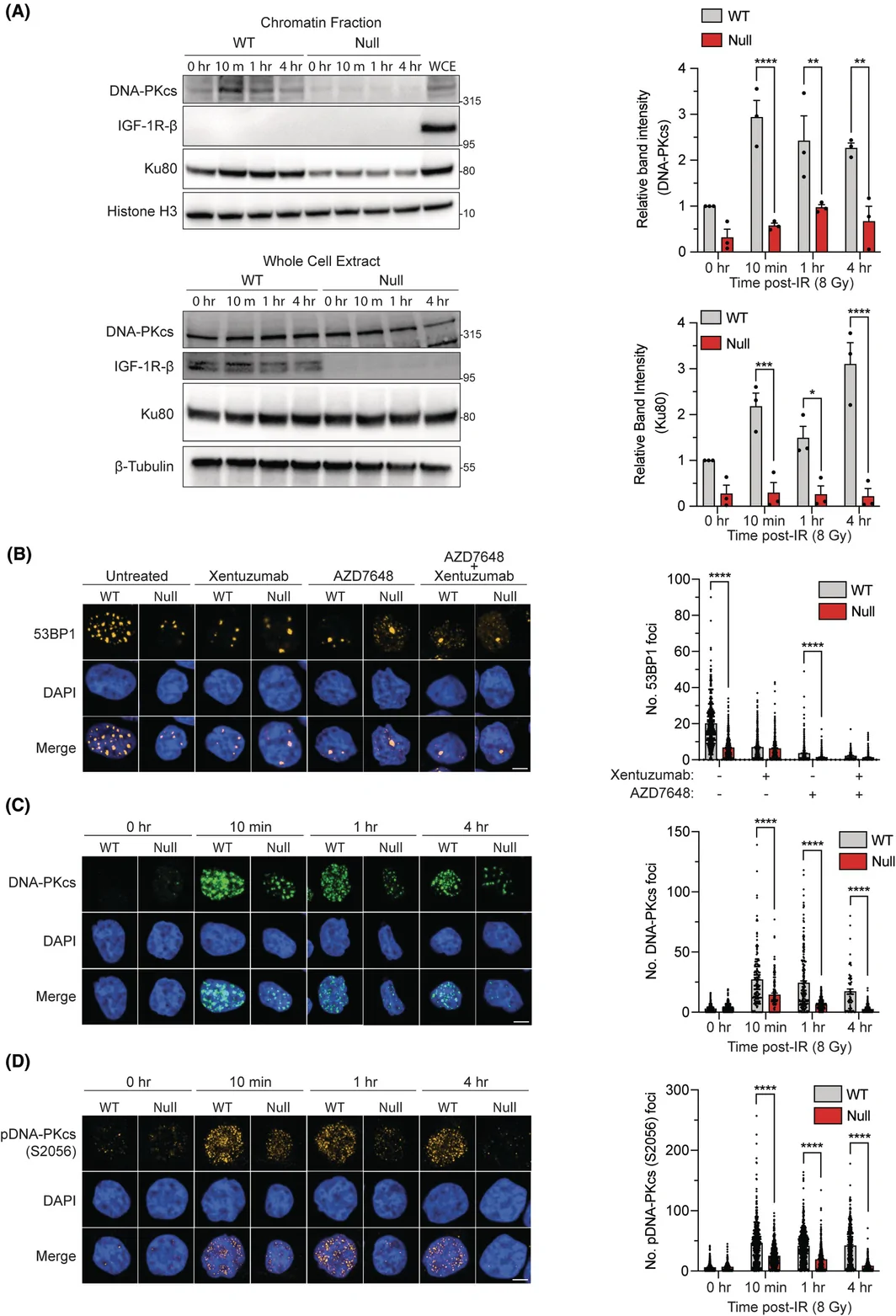
IGF‐1R status impacts DNA‐PKcs localisation to chromatin and non‐homologous end‐joining signalling in response to irradiation. (A) Chromatin fractionation of type 1 insulin‐like growth factor receptor (IGF1R)+/+ (wild‐type (WT)) and IGF1R−/− (null) clones pre‐ and post‐irradiation (IR) (8 Gy) at indicated time points. Left: Western blots are representative of three independent repeats. Right: Quantification of bands in three independent repeats (error bars represent ±SEM). DNA‐dependent protein kinase catalytic subunit (DNA‐PKcs) 10 min: ****P < 0.0001, 1 h: **P < 0.01, 4 h: **P < 0.01, Ku80 10 min: ***P < 0.001, 1 h: *P < 0.05, 4 h: ****P < 0.0001 by two‐way ANOVA. (B) Immunofluorescence staining of one IGF1R+/+ (WT) and one IGF1R−/− (null) clone pre‐ and 10 min post‐IR (8 Gy). Indicated cells were treated with DNAPKi (AZD7648) (1 μm), xentuzumab (1 μm) or in combination for 4 h prior to IR. Cells were stained for p53‐binding protein 1 (53BP1) (orange) and DAPI (blue) and foci were plotted (n = 3 independent experiments, error bars represent ±SEM). Untreated: ****P < 0.0001, AZD7648: ****P < 0.0001 by two‐way ANOVA. Scale bar = 10 μM. (C) Immunofluorescence staining of one IGF1R+/+ (WT) and one IGF1R−/− (null) clone after fixation at indicated time intervals after 8 Gy IR. Cells were stained for DNA‐PKcs (green) and DAPI (blue) and foci were plotted (n = 3 independent experiments, error bars represent ±SEM). 10 min: ****P < 0.0001, 1 h: ****P < 0.0001, 4 h: ****P < 0.0001 by two‐way ANOVA. Scale bar = 10 μM. (D) Immunofluorescence staining of one IGF1R+/+ (WT) and one IGF1R−/− (null) clone after fixation at indicated time intervals after 8 Gy IR. Cells were stained for pDNA‐PKcs (S2056) (orange) and DAPI (blue) and foci were plotted (n = 3 independent experiments, error bars represent ±SEM). 10 min: ****P < 0.0001, 1 h: ****P < 0.0001, 4 h: ****P < 0.0001 by two‐way ANOVA. Scale bar = 10 μM. Imaging was performed using the Opera Phenix Spinning Disk Confocal microscope and foci were analysed using Harmony High‐Content Imaging and Analysis software (Perkin Elmer).

Next, we investigated whether decreased DNA‐PKcs phosphorylation affects 53BP1 focus formation. Cells were treated for 4 h with solvent, xentuzumab (IGF‐1R ligand‐neutralising antibody), AZD7648 (DNAPKi [64]) or both, then irradiated and fixed at 10 min. Solvent‐treated *IGF1R* ^*−/−*^ cells had fewer 53BP1 foci than in *IGF1R* ^*+/+*^ cells (Fig. 3B), consistent with defective cNHEJ. Xentuzumab treatment reduced 53BP1 foci in *IGF1R* ^*+/+*^ cells to levels comparable to those in *IGF1R* ^*−/−*^ cells ± xentuzumab, while AZD7648 treatment decreased IR‐induced 53BP1 foci in *IGF1R* ^*+/+*^ cells as predicted. Notably, DNA‐PKcs inhibition in *IGF1R* ^*−/−*^ cells further reduced 53BP1 foci (Fig. 3B), likely reflecting reduced Ku80 recruitment to chromatin (Fig. 3A). This is consistent with studies showing that Ku80 can promote cNHEJ in the absence of functioning DNA‐PKcs [38]. In fact, Ku may be more important for DNA repair than DNA‐PKcs, given murine studies showing that *PRKDC* ^*−/−*^ mice display milder defects than *Ku* ^*−/−*^ mice [65, 66].

To complement this finding, we examined DNA‐PKcs focus formation post‐IR in CSK‐treated cells, a condition that removes soluble and weakly bound nuclear proteins to allow visualisation of proteins tightly associated with chromatin. As seen in Fig. 3A, we observed significantly decreased DNA‐PKcs foci in *IGF1R* ^*−/−*^ cells at each timepoint post‐IR (Fig. 3C), as well as decreased pS2056 DNA‐PKcs foci (Fig. 3D). Together, these data suggest that IGF‐1R loss impairs DNA‐PKcs chromatin recruitment and autophosphorylation, leading to compromised cNHEJ and radiosensitivity.

### Cancer cells defective in cNHEJ show increased MMEJ dependence and are sensitised to MMEJ inhibition

We then investigated whether the cNHEJ defect in *IGF1R* ^*−/−*^ cells leads to compensatory use of alternative repair pathways. Several reports suggest that in cells where cNHEJ is impaired, there is increased dependency on MMEJ, a more error‐prone repair mechanism [67, 68, 69]. To test MMEJ activity in prostate cancer models, we used a plasmid‐based end‐joining assay developed by the van Gent laboratory [28]. This requires transient transfection of linearised pDVG94 plasmid, followed by assessment of rejoined DNA by PCR amplification and restriction enzyme digest, with presence of a 120 bp product correlating with MMEJ activity. As a positive control, we treated *IGF1R* ^*+/+*^ cells with AZD7648 and detected an increase in the 120 bp product compared to control‐treated cells (Fig. 4A). This is consistent with other published data where this assay was used after disrupting the function of key cNHEJ proteins [70, 71, 72]. Interestingly, untreated *IGF1R* ^*−/−*^ cells already exhibited higher levels of the MMEJ product compared to *IGF1R* ^*+/+*^ cells, and no further increase was observed upon AZD7648 treatment (Fig. 4A), supporting the hypothesis that *IGF1R* ^*−/−*^ cells are more reliant on MMEJ.

**Fig. 4:**
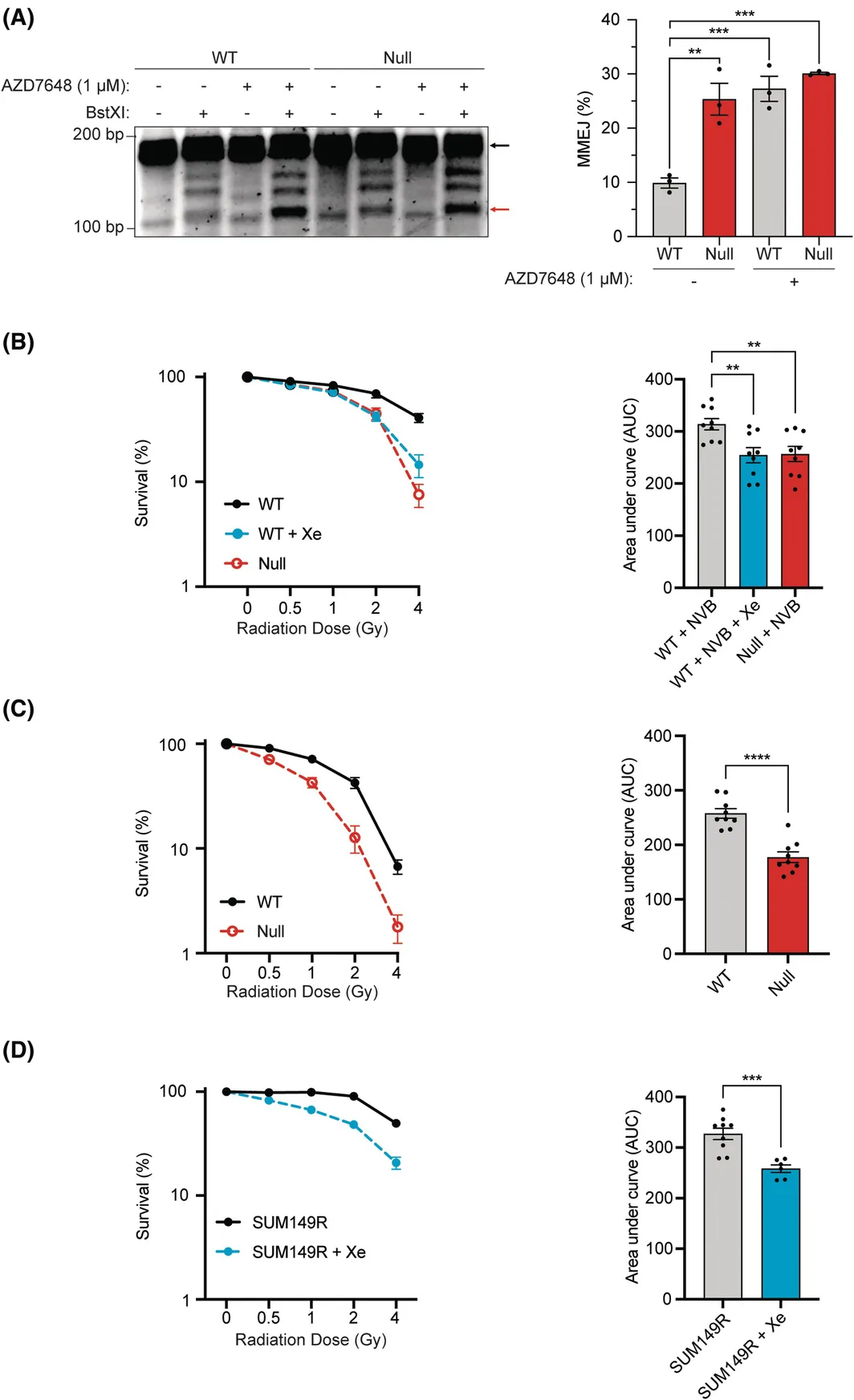
Defective IGF signalling sensitises cells to microhomology‐mediated end‐joining inhibitors. (A) Microhomology‐mediated end‐joining (MMEJ) repair reporter assay in type 1 insulin‐like growth factor receptor (IGF1R)+/+ (wild‐type (WT)) and IGF1R−/− (null) cells. Indicated cells were treated with DNAPKi (AZD7648) 4 h prior to transfection of linearised pDVG94 plasmid. The original plasmid was recovered and amplified before BstXI digestion. Quantification of BstXI‐digested 120 bp product intensity (red arrow)/200 bp product intensity (black arrow) produces MMEJ %. Left: Agarose gel representative of three independent repeats. Additional bands in digested samples likely represent minor imprecise end‐joining products resulting from microhomology‐dependent repair or partial restriction digestion, similar to other published reports [87, 88]. Right: Graph showing MMEJ % (n = 3 independent experiments, error bars represent ±SEM). Band intensity was measured using ImageJ. IGF1R+/+ vs IGF1R−/−: **P < 0.01, IGF1R+/+ vs IGF1R+/+ + AZD7648: ***P < 0.001, IGF1R+/+ vs IGF1R−/− + AZD7648: ***P < 0.001 by one‐way ANOVA. (B) Radiosensitivity assay examining the effect of DNA polymerase theta (POLθ) inhibition by novobiocin (NVB) in cells with differing IGF‐1R status. Cells were treated with NVB ± xentuzumab 4 h pre‐ionising radiation (IR) (at indicated doses) and counted after 10–13 days using the GelCount. Left: Graph showing cell survival as % unirradiated controls (one representative repeat of 3 independent biological repeats, error bars represent ±SEM). Area under curve: Unpaired t‐test: **P < 0.01 (n = 3 independent experiments, error bars represent ±SEM). Representative clonogenic assay dishes in Fig. S3B. (C) Radiosensitivity assay using one IGF1R+/+ and one IGF1R−/− clone after MMEJ inhibition by olaparib. Cells were treated with olaparib 4 h before IR at indicated doses and counted after 10–13 days using the GelCount. Left: Graph showing cell survival as % unirradiated controls (one representative repeat of 3 independent biological repeats, error bars represent ±SEM). Area under curve: Unpaired t‐test: ****P < 0.0001 (n = 3 independent experiments, error bars represent ±SEM). Representative clonogenic assay dishes in Fig. S3C. (D) Radiosensitivity assay in SUM149R cells treated with olaparib ± xentuzumab. Drugs were added 4 h pre‐IR at indicated doses and counted after 10–13 days using the GelCount. Left: Graph showing cell survival as % unirradiated controls (one representative repeat of 3 independent biological repeats, error bars represent ±SEM). Area under curve: Unpaired t‐test: ***P < 0.001 (n = 3 independent experiments, error bars represent ±SEM). Representative clonogenic assay dishes in Fig. S3D.

We then tested whether this MMEJ reliance introduces therapeutic vulnerabilities. MMEJ is initiated by rapid recruitment of poly‐ADP ribose polymerase‐1 (PARP‐1) to break sites [73], which also influences repair pathway choice and chromatin structure. The sensitivity of HR‐deficient tumours to PARP inhibition [74, 75] has been leveraged clinically and similar logic could apply here.

To explore this, we targeted two MMEJ effectors – POLθ and PARP‐1 – in IGF‐1R‐deficient settings. Clonogenic survival assays were performed after pre‐treatment with POLθi and subsequent radiation treatment. POLθi sensitivity was significantly increased post‐IR at all radiation doses in cells with both acute inhibition (*IGF1R* ^*+/+*^ + xentuzumab) and chronic loss (*IGF1R* ^*−/−*^) of IGF‐1R compared to *IGF1R* ^*+/+*^ cells (Fig. 4B, Fig. S3B, Table S5), consistent with our hypothesis that IGF‐1R loss causes defective cNHEJ and over‐reliance on MMEJ.

Next, we tested whether PARPi could further sensitise *IGF1R* ^*−/−*^ cells to IR. Clonogenic survival assays were performed on *IGF1R* ^*+/+*^ and *IGF1R* ^*−/−*^ cells pre‐treated with PARPi, followed by irradiation. *IGF1R* ^*−/−*^ cells showed increased radiosensitivity after PARPi treatment (Fig. 4C, Fig. S3C, Table S5) further suggesting defective cNHEJ. While PARPi treatment has proven highly successful in the treatment of HR‐deficient tumours, resistance does develop [76, 77, 78, 79]. We hypothesised that IGF‐1R inhibition could overcome this.

Using SUM149 *BRCA1* ^*−/−*^ (SUM149PT) breast cancer cells with a *BRCA1* revertant mutation (SUM149R) that leads to PARPi resistance, we investigated whether inhibition of IGF‐1R signalling could resensitise PARPi‐resistant cells to PARP‐1 inhibition. We performed clonogenic assays with SUM149R cells in the presence of PARPi ± xentuzumab at increasing radiation doses. At increasing doses of IR, olaparib‐resistant cells treated with xentuzumab displayed increased sensitivity (Fig. 4D, Fig. S3D, Table S5). This apparent ability for IGF‐1R inhibition to resensitise PARPi‐resistant cells suggested a role for IGF‐1R in PARPi resistance, or more broadly in MMEJ pathway resistance.

## Discussion

In this study, we identify IGF‐1R as a previously underappreciated regulator of DSB repair, acting through control of DNA‐PKcs recruitment and activity at chromatin. Loss of IGF‐1R compromises cNHEJ, resulting in defective DNA‐PKcs autophosphorylation, impaired 53BP1 signalling, and delayed resolution of IR‐induced DNA damage. As a consequence, IGF1R‐deficient cells exhibit increased reliance on alternative end‐joining pathways, particularly MMEJ, which creates a therapeutically exploitable vulnerability.

Our data demonstrate that IGF‐1R loss markedly reduces DNA‐PKcs localisation to chromatin following irradiation, accompanied by loss of S2056 autophosphorylation. Consistently, chromatin fractionation showed markedly reduced recruitment of both DNA‐PKcs and its binding partner Ku80 in IGF1R^−/−^ cells following IR (Fig. 3A), highlighting a tangible loss of NHEJ machinery at DNA break sites. This phenotype is consistent across independent IGF1R knockout clones and is partially recapitulated by acute ligand neutralisation (notably reduced 53BP1 foci following IR), supporting a requirement for intact IGF signalling rather than long‐term adaptive effects. Importantly, ATM signalling is largely preserved, as evidenced by retained ATM autophosphorylation and no significant reduction in KAP1 phosphorylation, indicating that the primary defect lies within the DNA‐PK–dependent arm of the DSB response.

Mechanistically, our findings place IGF‐1R upstream of DNA‐PKcs recruitment rather than as a structural component of the chromatin‐bound repair complex. Although we detect DNA‐PKcs as a high‐confidence interactor of IGF‐1R by immunoprecipitation and mass spectrometry, IGF‐1R itself is not retained in chromatin fractions following damage. This suggests a model in which IGF‐1R facilitates DNA‐PKcs mobilisation or stabilisation prior to or during engagement with DNA ends, without remaining chromatin‐associated. Such a mechanism is compatible with previous reports describing nuclear translocation of activated IGF‐1R [54, 55, 56] and its interaction with DNA‐PKcs in response to genotoxic stress [14, 80], while also explaining the absence of IGF‐1R from detergent‐resistant chromatin extracts. Whether this regulation is mediated through transient protein–protein interactions, modulation of DNA‐PKcs post‐translational modification, or indirect signalling effects remains to be resolved. We also note emerging evidence for non‐nuclear roles of DNA‐PKcs [81, 82]; while we have not dissected cytoplasmic versus nuclear IGF‐1R–DNA‐PKcs interactions here, this may represent an additional layer of regulation in specific contexts.

Functionally, impaired cNHEJ in IGF1R‐deficient cells is accompanied by a shift toward MMEJ. Using a plasmid‐based end‐joining assay, we observe increased microhomology‐dependent repair products at baseline in IGF1R‐null cells, with no further increase upon DNA‐PKcs inhibition, consistent with pathway epistasis. This rewiring of DSB repair creates dependency on MMEJ effectors, reflected by increased radio‐sensitisation by PARP‐1 and polymerase‐θ inhibition. These findings align with broader evidence that suppression of cNHEJ promotes compensatory engagement of error‐prone repair pathways, and extend this concept to IGF‐1R–dependent regulation of repair pathway choice [83, 84].

The therapeutic implications of these findings are twofold. First, IGF‐1R inhibition enhances radiosensitivity by directly compromising DNA‐PKcs‐dependent repair, supporting its potential use as a radiosensitising strategy in tumours with intact HR. Second, and more unexpectedly, IGF‐1R inhibition re‐sensitises PARPi‐resistant cells to IR when combined with Olaparib in a BRCA1‐revertant, PARPi‐resistant breast cancer model. This suggests that IGF‐1R contributes to repair plasticity underlying acquired PARPi resistance, potentially by maintaining sufficient cNHEJ capacity to tolerate PARP‐induced lesions. In this context, IGF‐1R inhibition may limit compensatory repair routes and re‐establish therapeutic vulnerability.

Notably, prior studies have reported functional interplay between IGF‐1R inhibition and PARP inhibition in several tumour models [85, 86], consistent with the concept that IGF axis modulation can expose or exacerbate DNA repair dependencies. Our data extend this by linking IGF‐1R status to DNA‐PKcs chromatin recruitment and repair pathway choice, providing a mechanistic basis for rational combinations with PARPi in defined contexts. While we do not exclude additional contributions from IGF‐dependent signalling pathways, the consistency of the DNA‐PKcs localisation defect across genetic and pharmacological models supports a direct role for IGF‐1R in regulating cNHEJ competence.

Taken together, these data support a model in which IGF‐1R sustains efficient DNA‐PKcs‐mediated end‐joining, thereby constraining reliance on error‐prone repair pathways. Loss or inhibition of IGF‐1R disrupts this balance, sensitising cells to DNA damage and exposing vulnerabilities to PARP and POLθ inhibition. These findings provide a rationale for stratifying tumours by IGF‐1R status when considering combinations of radiotherapy with DNA repair inhibitors, particularly in settings of PARP inhibitor resistance.

## Conclusion

Currently, POLθi are in clinical development for use against PARPi‐resistant cancers. Our data suggest that combination treatment with IGF‐1R and POLθi resensitises PARPi‐resistant tumours to IR, particularly in IGF‐1R deficient prostate cancer. These results are consistent with a cNHEJ defect in *IGF1R* ^*−/−*^ cells and provide a mechanistic basis for impaired DSB repair identified by our group previously [14]. Altogether, we show that IGF‐1R status affects DNA‐PKcs localisation and function, leading to increased dependency on MMEJ. Finally, we posit that IGF‐1R inhibition may be clinically exploitable, especially in tumours resistant to PARPi or POLθi.

## Conflict of interest

The authors declare no conflicts of interest.

## Author contributions

Conceptualisation: VMM, MOE. Methodology: MOE, VMM, JVM. Data analysis: MOE. Writing and editing: MOE, VMM, WN. Data acquisition: MOE, JVM. Supervision: VMM, WN.

## Supporting information


**Fig. S1.** Creation of IGF‐1R null 22Rv1 clones. (A) Representative clonogenic assay dishes of DU145 cells transfected with control (siControl) or type 1 insulin‐like growth factor receptor (IGF‐1R) (siIGF‐1R) siRNAs subjected to increasing doses of ionising radiation (IR) after 10–13 days, as described in Fig. 1A. (B) Representative clonogenic assay dishes of 22Rv1 cells transfected with control (siControl) or IGF‐1R (siIGF‐1R) siRNAs subjected to increasing doses of IR after 10–13 days, as described in Fig. 1A. (C) IGF‐1R enzyme‐linked immunosorbent assay (ELISA) for characterisation of candidate IGF1R−/− clones. Absorbance (450 nm) was measured on a POLARstar OMEGA plate reader and expressed relative to mean IGF1R+/+ values. (D) Western blot of candidate IGF1R−/− clones with relative fluorescence unit (RFU) < 0.15 relative to mean IGF1R+/+ values in A. (E) Band intensities were quantified using ImageJ (n = 3 independent experiments, error bars represent ±SEM). Graphs display levels of indicated phospho‐proteins relative to IGF1R+/+ cells. (F) Sanger sequencing of genomic DNA from two IGF1R−/− clones. Black dashed lines indicate guide RNA (gRNA) cut site.

**Fig. S2.** Characterisation of IGF‐1R null 22Rv1 clones. Phospho‐receptor dose response enzyme‐linked immunosorbent assays (ELISAs) performed using antibodies to (A) phospho‐Y1135/6 type 1 insulin‐like growth factor receptor (IGF‐1R) for insulin‐like growth factor 1 (IGF‐1) and (B) phospho‐Y1150/1151 insulin receptor (INSR) for insulin. Absorbance (450 nm) was measured on a POLARstar OMEGA plate reader. (C) Proliferation rate of parental 22Rv1 cells, two IGF1R+/+ and IGF1R−/− clones. Confluency was measured using the Incucyte Live‐Cell Analysis System (Sartorius). (D) Clonogenic survival of untreated/unirradiated IGF1R+/+ and IGF1R−/− clones. Colonies were stained after 10–13 days and counted using the GelCount. (E) Representative clonogenic assay dishes of survival assay shown in Fig. 1B subjected to increasing doses of ionising radiation (IR) after 10–13 days. (F) Quantification of p53‐binding protein 1 (53BP1) immunofluorescence (IF) staining of two IGF1R+/+ and two IGF1R−/− clones after fixation 10 min post‐IR (8 Gy). Foci were plotted (n = 3 independent experiments, error bars represent ±SEM).

**Fig. S3.** Clonogenic sensitivity to microhomology‐mediated end‐joining and poly (ADP‐ribose) polymerase inhibitors. (A) Validation of nuclear type 1 insulin‐like growth factor receptor (IGF‐1R) interaction with DNA‐dependent protein kinase catalytic subunit (DNA‐PKcs) with and without irradiation (IR) by western blot. Cells were fractionated, and nuclear lysates were immunoprecipitated with antibodies for IgG or IGF‐1Rβ and analysed alongside nuclear (N), cytoplasmic (C) and whole cell extract (WCE) input controls. Subcellular fractionation was confirmed by blotting for nuclear marker lamin A/C and cytosolic marker (β‐tubulin). (B) Representative clonogenic assay dishes from radiosensitivity assays examining the effect of DNA polymerase theta (POLθ) inhibition by novobiocin (NVB) in cells with differing IGF‐1R status. (C) Representative clonogenic assay dishes from radiosensitivity assays using one IGF1R+/+ and one IGF1R−/− clone after poly (ADP‐ribose) polymerase 1 (PARP1) inhibition by olaparib. (D) Representative clonogenic assay dishes from radiosensitivity assays in SUM149R cells treated with olaparib ± xentuzumab.

**Table S1.** Primer list. All sequences shown 5′ – 3′.

**Table S2.** Antibodies used in this study. IF, immunofluorescence; WB, western blot; IP, immunoprecipitation; CST, Cell Signalling Technology.

**Table S3.** Dose enhancement ratios for Fig. 1A,B. Dose enhancement ratios (DERs) are calculated relative to siControl or type 1 insulin‐like growth factor receptor (*IGF1R*)^*+/+*^ cells.

**Table S4.** List of nuclear IGF‐1R interactome hits identified by mass spectrometry. Full list of nuclear type 1 insulin‐like growth factor receptor (IGF‐1R) interactome hits identified by mass spectrometry in each indicated *IGF1R* ^*+/+*^ (WT) or *IGF1R* ^*−/−*^ (null) sample. Table shows Accession Number for each identified protein and protein name/description including gene code. For each sample in which peptides for indicated protein could be identified the table shows Protein Score (Prot Score ‐ sum of the ion score of all identified peptides), Peptide number (The total number of distinct peptide sequences identified in the protein group) and Unique Peptide number (The number of identified peptide sequences that are unique to a protein group). Columns in which no data is present indicate that the relevant peptide was not detected in that sample.

**Table S5.** Dose enhancement ratios for Fig. 4B–D. Dose enhancement ratios (DERs) are calculated relative to type 1 insulin‐like growth factor receptor (*IGF1R*)^*+/+*^ (WT) cells (Fig. 4B,C) or SUM149R (Fig. 4D).

## Data Availability

The data that support the findings of this study are available from the corresponding author [moe23@cam.ac.uk] upon reasonable request.
