# Supplementary material for: Loss of IGF‐1R impairs DNA‐PKcs recruitment to chromatin leading to defective end‐joining
Source: Mol Oncol. 2026 May 7;20(11):2876–95. doi: 10.1002/1878-0261.70266 (PMC13398348; doi:10.1002/1878-0261.70266)
Supplement: Supplementary file 1 [file MOL2-20-2876-s001.docx]

| **Use** | **Antibody target** | **Supplier** | **Catalogue no** |
| --- | --- | --- | --- |
| IF | 53BP1 | Novus Biologicals | NB100-304 |
| WB | ATM | CST | #9272 |
| WB | β-tubulin | Sigma Aldrich | T6074 |
| IP, WB | DNA-PKcs | Sigma Aldrich | MABC1236 |
| IF | DNA-PKcs | Abcam | Ab133516 |
| WB | Histone H3 | CST | #4499 |
| IP, WB, ELISA | IGF-1Rβ | CST | #3027 |
| WB | KAP-1 | CST | #4123 |
| WB | Ku80 | Thermo Fisher Scientific | MA5-12933 |
| IP | Lamin A/C | Abcam | Ab224816 |
| WB | pATM-S1981 | Abcam | Ab81292 |
| IF, WB | pDNA-PKcs-S2056 | Abcam | Ab18192 |
| WB | pKAP-1-S824 | Abcam | Ab70369 |
| IF | pH2AX-S139 (γH2AX) | Millipore | 05-636 |
| IF | Alexa Fluor 488-conjugated goat anti-mouse | Thermo Fisher Scientific | A20181 |
| ELISA | pY1135/1136 IGF-1R  pY1150/Y1151 INSR | CST | #3024 |
| IF | Alexa Fluor 594-conjugated goat anti-rabbit | Thermo Fisher Scientific | A11012 |
| IP, WB | Anti-mouse IgG-HRP | Dako | P0447 |
| IP, WB, ELISA | Anti-rabbit IgG-HRP | Dako | P0448 |

**Supplementary Table S2. Antibodies used in this study.** IF, immunofluorescence; WB, Western blot; IP, immunoprecipitation; CST, Cell Signaling Technology.
