# Supplementary material for: Loss of IGF‐1R impairs DNA‐PKcs recruitment to chromatin leading to defective end‐joining
Source: Mol Oncol. 2026 May 7;20(11):2876–95. doi: 10.1002/1878-0261.70266 (PMC13398348; doi:10.1002/1878-0261.70266)
Supplement: Supplementary file 2 [file MOL2-20-2876-s002.docx]

| **Primer** | **Primer sequence** | **Function** |
| --- | --- | --- |
| IGF1R_Exon2_gRNAf | CACCGGCATCGACATCCGCAACGA | gRNAs to target exon 2 of *IGF1R* using CRISPR |
| IGF1R_Exon2_gRNAr | AAACTCGTTGCGGATGTCGATGCC |  |
| DAR5 | TGCTTCCGGCTCGTATGTTGGTTGGAAT | Primers for amplification of PCR products in MMEJ assay as described^1^ |
| FM30 | CTCCATTTTAGCTTCCTTAGCTCCTG |  |

**Supplementary Table S1. Primer list.** All sequences shown 5’ – 3’.
