# Supplementary material for: Loss of IGF‐1R impairs DNA‐PKcs recruitment to chromatin leading to defective end‐joining
Source: Mol Oncol. 2026 May 7;20(11):2876–95. doi: 10.1002/1878-0261.70266 (PMC13398348; doi:10.1002/1878-0261.70266)
Supplement: Supplementary file 4 [file MOL2-20-2876-s004.docx]

| **Radiation dose (Gy)** | | **0.5** | | **1** | | **2** | | **4** | |
| --- | --- | --- | --- | --- | --- | --- | --- | --- | --- |
|  | | **Average** | **SEM** | **Average** | **SEM** | **Average** | **SEM** | **Average** | **SEM** |
| **Figure 4B** | **WT + Xe** | 1.10 | 0.16 | 1.20 | 0.19 | 1.67 | 0.24 | 19.59 | 28.02 |
|  | **Null** | 1.08 | 0.17 | 1.19 | 0.27 | 1.70 | 0.61 | 19.56 | 28.72 |
| **Figure 4C** | **Null** | 1.29 | 0.09 | 1.79 | 0.53 | 6.00 | 4.81 | 4.91 | 3.55 |
| **Figure 4D** | **SUM149R + Xe** | 1.28 | 0.06 | 1.67 | 0.24 | 2.50 | 0.34 | 4.09 | 1.45 |

**Supplementary Table S5. Dose enhancement ratios for figures 4B-D.**

Dose enhancement ratios (DERs) are calculated rtype 1 insulin-like growth factor receptor (*IGF1R*)*^+/+^* cells (Figure 4B-C) or SUM149R (Figure 4D).
