# Supplementary figures and images for: Loss of IGF‐1R impairs DNA‐PKcs recruitment to chromatin leading to defective end‐joining
Source: Mol Oncol. 2026 May 7:10.1002/1878-0261.70266. Online ahead of print. doi: 10.1002/1878-0261.70266 (PMC13398348; doi:10.1002/1878-0261.70266)

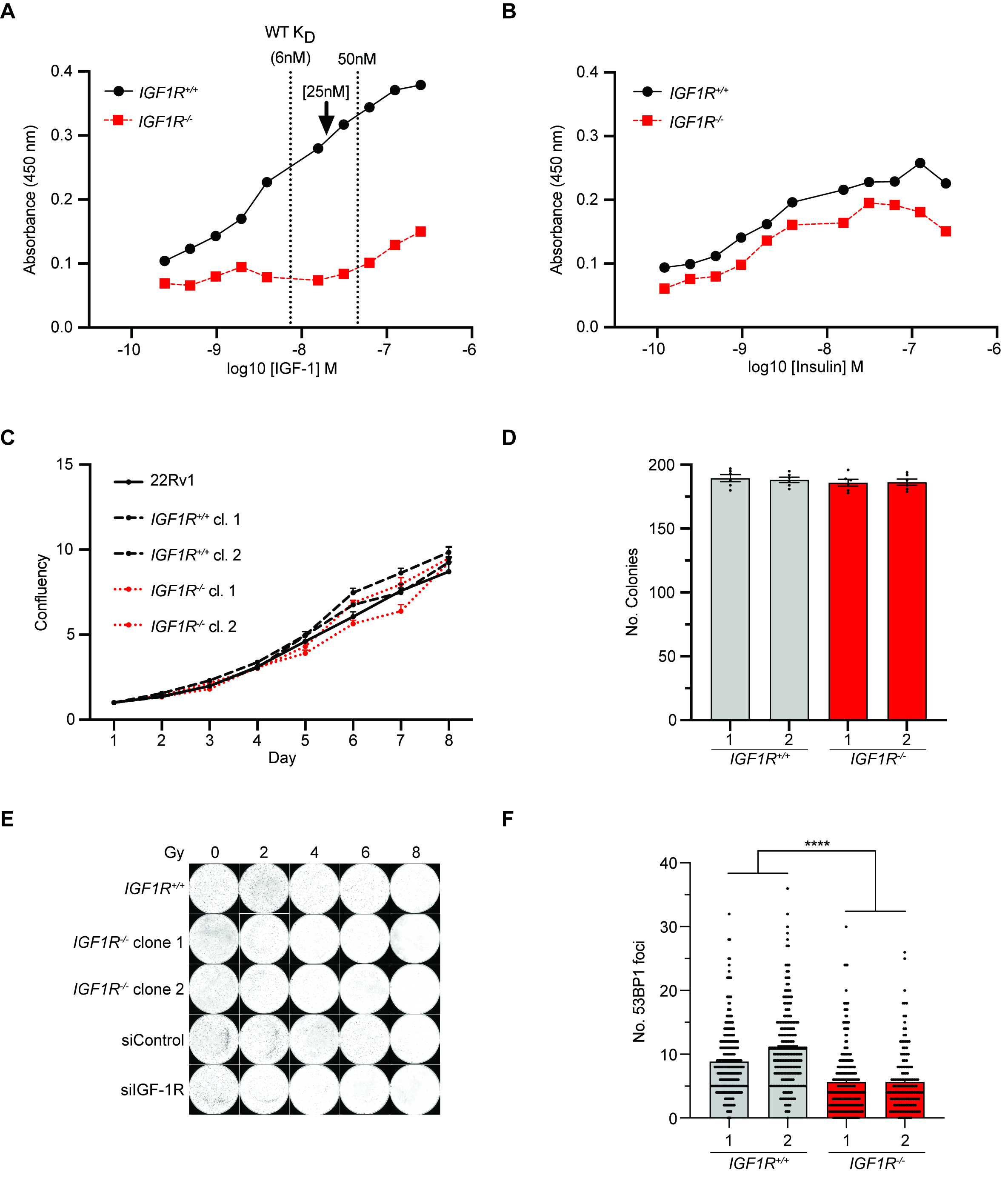

Supplement: Supplementary file 1 — Fig. S1. Creation of IGF‐1R null 22Rv1 clones. (A) Representative clonogenic assay dishes of DU145 cells transfected with control (siControl) or type 1 insulin‐like growth factor receptor (IGF‐1R) (siIGF‐1R) siRNAs subjected to increasing doses of ionising radiation (IR) after 10–13 days, as described in Fig. 1A. (B) Representative clonogenic assay dishes of 22Rv1 cells transfected with control (siControl) or IGF‐1R (siIGF‐1R) siRNAs subjected to increasing doses of IR after 10–13 days, as described in Fig. 1A. (C) IGF‐1R enzyme‐linked immunosorbent assay (ELISA) for characterisation of candidate IGF1R−/− clones. Absorbance (450 nm) was measured on a POLARstar OMEGA plate reader and expressed relative to mean IGF1R+/+ values. (D) Western blot of candidate IGF1R−/− clones with relative fluorescence unit (RFU) < 0.15 relative to mean IGF1R+/+ values in A. (E) Band intensities were quantified using ImageJ (n = 3 independent experiments, error bars represent ±SEM). Graphs display levels of indicated phospho‐proteins relative to IGF1R+/+ cells. (F) Sanger sequencing of genomic DNA from two IGF1R−/− clones. Black dashed lines indicate guide RNA (gRNA) cut site. [file MOL2-9999-0-s008.tif]

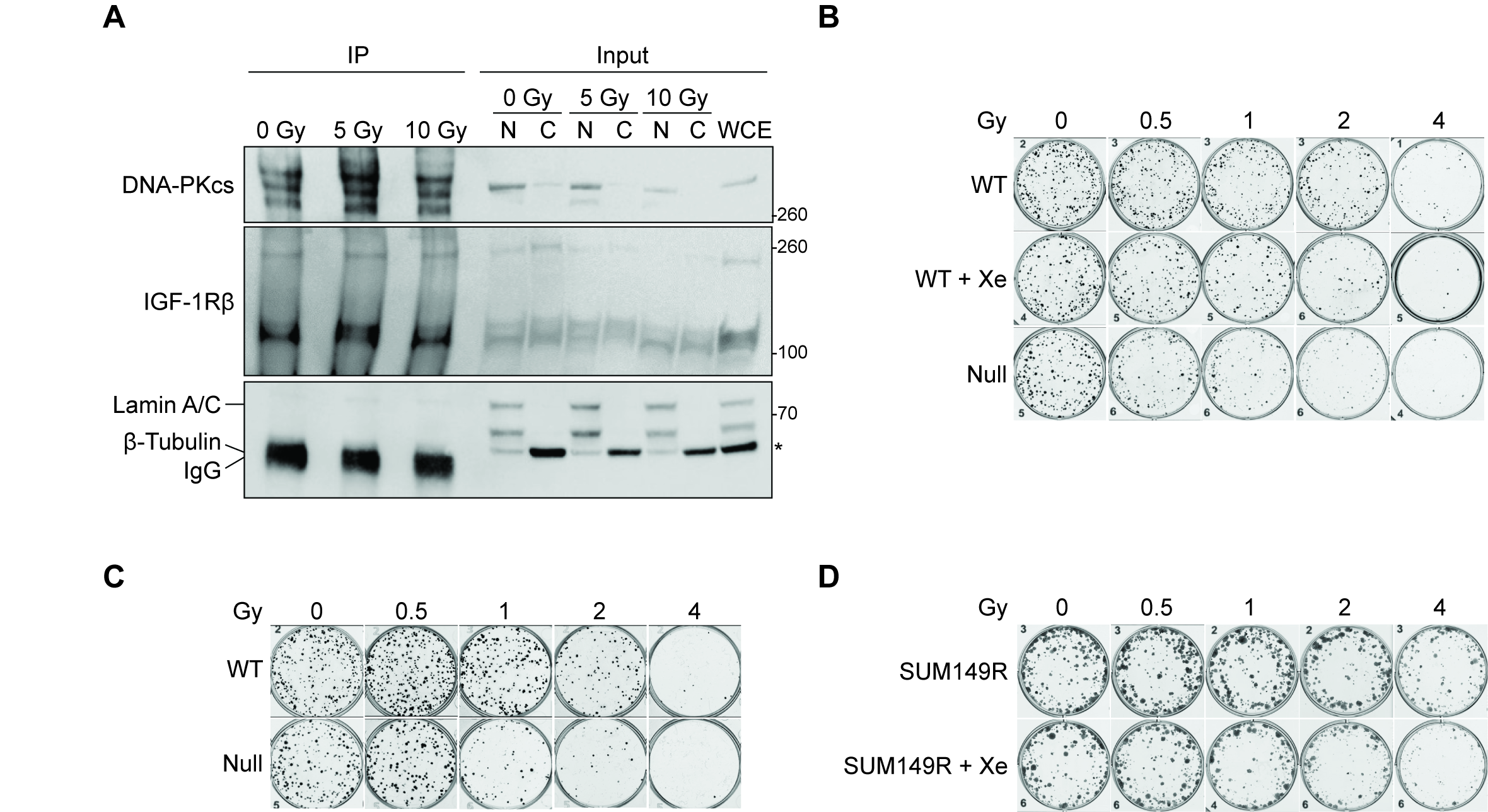

Supplement: Supplementary file 2 — Fig. S2. Characterisation of IGF‐1R null 22Rv1 clones. Phospho‐receptor dose response enzyme‐linked immunosorbent assays (ELISAs) performed using antibodies to (A) phospho‐Y1135/6 type 1 insulin‐like growth factor receptor (IGF‐1R) for insulin‐like growth factor 1 (IGF‐1) and (B) phospho‐Y1150/1151 insulin receptor (INSR) for insulin. Absorbance (450 nm) was measured on a POLARstar OMEGA plate reader. (C) Proliferation rate of parental 22Rv1 cells, two IGF1R+/+ and IGF1R−/− clones. Confluency was measured using the Incucyte Live‐Cell Analysis System (Sartorius). (D) Clonogenic survival of untreated/unirradiated IGF1R+/+ and IGF1R−/− clones. Colonies were stained after 10–13 days and counted using the GelCount. (E) Representative clonogenic assay dishes of survival assay shown in Fig. 1B subjected to increasing doses of ionising radiation (IR) after 10–13 days. (F) Quantification of p53‐binding protein 1 (53BP1) immunofluorescence (IF) staining of two IGF1R+/+ and two IGF1R−/− clones after fixation 10 min post‐IR (8 Gy). Foci were plotted (n = 3 independent experiments, error bars represent ±SEM). [file MOL2-9999-0-s001.tif]

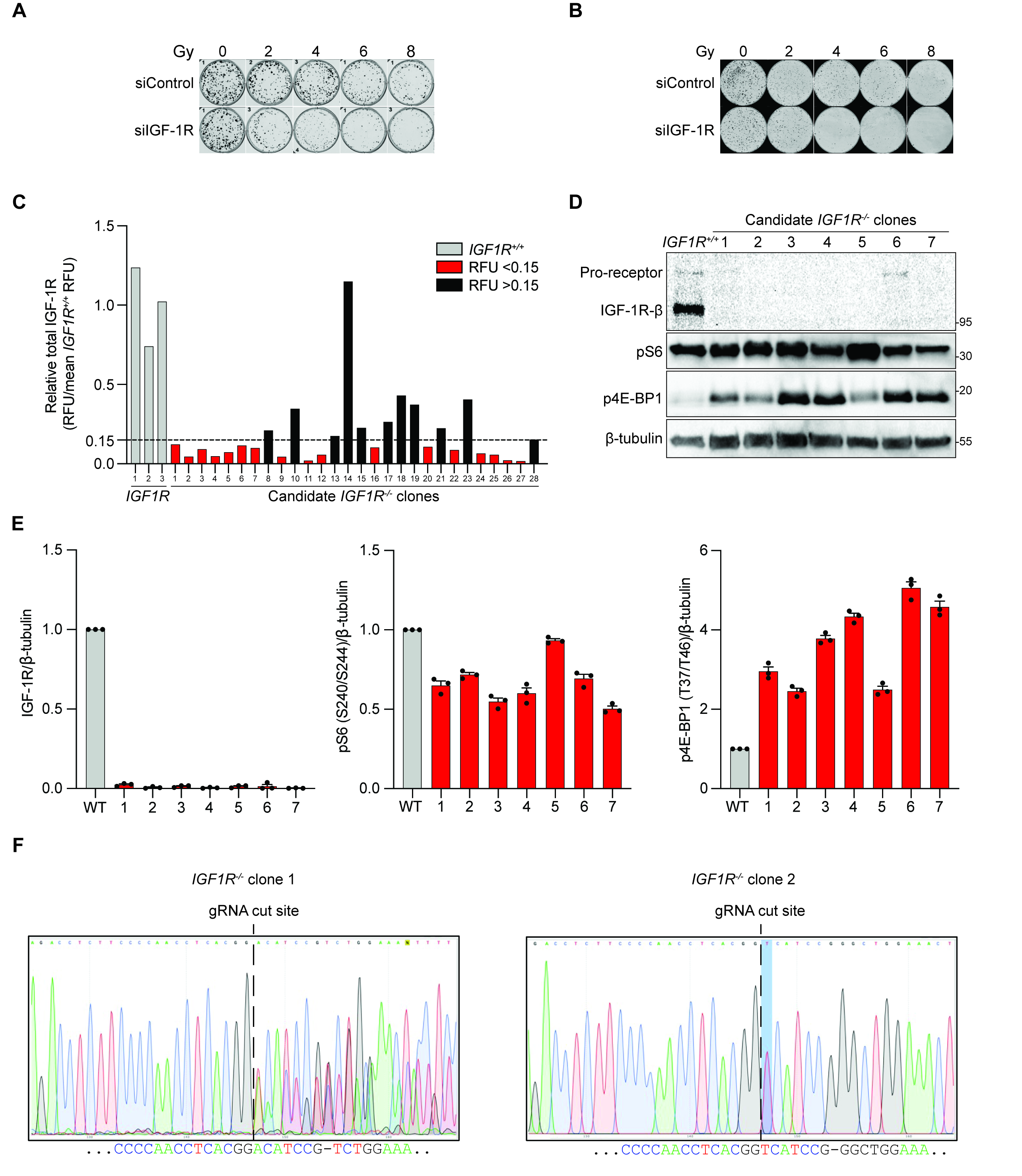

Supplement: Supplementary file 3 — Fig. S3. Clonogenic sensitivity to microhomology‐mediated end‐joining and poly (ADP‐ribose) polymerase inhibitors. (A) Validation of nuclear type 1 insulin‐like growth factor receptor (IGF‐1R) interaction with DNA‐dependent protein kinase catalytic subunit (DNA‐PKcs) with and without irradiation (IR) by western blot. Cells were fractionated, and nuclear lysates were immunoprecipitated with antibodies for IgG or IGF‐1Rβ and analysed alongside nuclear (N), cytoplasmic (C) and whole cell extract (WCE) input controls. Subcellular fractionation was confirmed by blotting for nuclear marker lamin A/C and cytosolic marker (β‐tubulin). (B) Representative clonogenic assay dishes from radiosensitivity assays examining the effect of DNA polymerase theta (POLθ) inhibition by novobiocin (NVB) in cells with differing IGF‐1R status. (C) Representative clonogenic assay dishes from radiosensitivity assays using one IGF1R+/+ and one IGF1R−/− clone after poly (ADP‐ribose) polymerase 1 (PARP1) inhibition by olaparib. (D) Representative clonogenic assay dishes from radiosensitivity assays in SUM149R cells treated with olaparib ± xentuzumab. [file MOL2-9999-0-s003.tif]
