# Supplementary material for: Loss of IGF‐1R impairs DNA‐PKcs recruitment to chromatin leading to defective end‐joining
Source: Mol Oncol. 2026 May 7:10.1002/1878-0261.70266. Online ahead of print. doi: 10.1002/1878-0261.70266 (PMC13398348; doi:10.1002/1878-0261.70266)
Supplement: Supplementary file 6 — Table S3. Dose enhancement ratios for Fig. 1A,B. Dose enhancement ratios (DERs) are calculated relative to siControl or type 1 insulin‐like growth factor receptor (IGF1R) +/+ cells. [file MOL2-9999-0-s005.docx]

| **Radiation dose (Gy)** | | **2** | | **4** | | **6** | | **8** | |
| --- | --- | --- | --- | --- | --- | --- | --- | --- | --- |
|  | | **Average** | **SEM** | **Average** | **SEM** | **Average** | **SEM** | **Average** | **SEM** |
| **Figure 1A** | **DU145 siIGF-1R** | 2.40 | 0.10 | 3.11 | 1.49 | 4.73 | 2.40 | 8.19 | 6.03 |
|  | **22Rv1 siIGF-1R** | 1.26 | 0.15 | 3.82 | 0.26 | 2.78 | 0.57 | 22.03 | 6.96 |
| **Figure 1B** | ***IGF1R^-/-^* cl. 1** | 1.34 | 0.03 | 2.70 | 0.35 | 4.97 | 2.89 | 15.49 | 6.33 |
|  | ***IGF1R^-/-^* cl. 2** | 1.82 | 0.27 | 2.06 | 0.30 | 2.63 | 1.11 | 16.64 | 15.83 |
|  | **siIGF-1R** | 0.97 | 0.10 | 1.41 | 0.10 | 2.11 | 0.24 | 4.86 | 2.00 |

**Supplementary Table S3. Dose enhancement ratios for figures 1A-B.** Dose enhancement ratios (DERs) are calculated relative to siControl or type 1 insulin-like growth factor receptor (*IGF1R*)*^+/+^* cells.
