# Supplementary material for: Loss of IGF‐1R impairs DNA‐PKcs recruitment to chromatin leading to defective end‐joining
Source: Mol Oncol. 2026 May 7:10.1002/1878-0261.70266. Online ahead of print. doi: 10.1002/1878-0261.70266 (PMC13398348; doi:10.1002/1878-0261.70266)
Supplement: Supplementary file 7 — Table S4. List of nuclear IGF‐1R interactome hits identified by mass spectrometry. Full list of nuclear type 1 insulin‐like growth factor receptor (IGF‐1R) interactome hits identified by mass spectrometry in each indicated IGF1R +/+ (WT) or IGF1R −/− (null) sample. Table shows Accession Number for each identified protein and protein name/description including gene code. For each sample in which peptides for indicated protein could be identified the table shows Protein Score (Prot Score ‐ sum of the ion score of all identified peptides), Peptide number (The total number of distinct peptide sequences identified in the protein group) and Unique Peptide number (The number of identified peptide sequences that are unique to a protein group). Columns in which no data is present indicate that the relevant peptide was not detected in that sample. [file MOL2-9999-0-s006.docx]

**Supplementary Table S4. List of nuclear IGF-1R interactome hits identified by mass spectrometry.**

Full list of nuclear type 1 insulin-like growth factor receptor (IGF-1R) interactome hits identified by mass spectrometry in each indicated *IGF1R^+/+^* (WT) or *IGF1R^-/-^* (null) sample. Table shows Accession Number for each identified protein and protein name/description including gene code. For each sample in which peptides for indicated protein could be identified the table shows Protein Score (Prot Score - sum of the ion score of all identified peptides), Peptide number (The total number of distinct peptide sequences identified in the protein group) and Unique Peptide number (The number of identified peptide sequences that are unique to a protein group). Columns in which no data is present indicate that the relevant peptide was not detected in that sample.

|  |  | **WT 1** | | | **WT 2** | | | **Null 1** | | | **Null 2** | | |
| --- | --- | --- | --- | --- | --- | --- | --- | --- | --- | --- | --- | --- | --- |
| **Acc**  **Number** | **Description** | **Prot score**  **(-10logP)** | **Peptide No.** | **Unique**  **Peptide No.** | **Prot score**  **(-10logP)** | **Peptide No.** | **Unique**  **Peptide No.** | **Prot score**  **(-10logP)** | **Peptide No.** | **Unique Peptide No.** | **Prot score**  **(-10logP)** | **Peptide No.** | **Unique Peptide No.** |
| P78527 | DNA-dependent protein kinase catalytic subunit GN=PRKDC | 282 | 33 | 33 | 361.51 | 79 | 79 | 125.84 | 8 | 8 | 158.65 | 11 | 11 |
| P52272 | Heterogeneous nuclear ribonucleoprotein M GN=HNRNPM | 333.25 | 32 | 32 | 313.07 | 27 | 27 | 156.59 | 13 | 13 | 69.83 | 2 | 2 |
| Q08211 | ATP-dependent RNA helicase A GN=DHX9 | 242.76 | 20 | 20 | 224.11 | 18 | 18 | 71.54 | 2 | 2 | 101.07 | 4 | 4 |
| P10809 | 60 kDa heat shock protein mitochondrial GN=HSPD1 | 251.68 | 19 | 19 | 164.65 | 12 | 12 | 90.52 | 5 | 5 | 89.36 | 3 | 3 |
| P38646 | Stress-70 protein mitochondrial GN=HSPA9 | 265.78 | 18 | 18 | 120.39 | 4 | 4 | 164.94 | 9 | 9 | 82.66 | 4 | 4 |
| P11142 | Heat shock cognate 71 kDa protein GN=HSPA8 | 232.84 | 19 | 15 | 212.31 | 16 | 14 | 147.76 | 9 | 8 | 61.45 | 2 | 2 |
| P17844 | Probable ATP-dependent RNA helicase DDX5 GN=DDX5 | 246.9 | 19 | 15 | 188.49 | 14 | 12 | 135.51 | 9 | 7 | 70.21 | 2 | 2 |
| P25705 | ATP synthase subunit alpha mitochondrial GN=ATP5F1A | 240.48 | 12 | 12 | 206.58 | 9 | 9 | 157.17 | 8 | 7 | 104.54 | 4 | 4 |
| P23396 | 40S ribosomal protein S3 GN=RPS3 | 224.47 | 12 | 12 | 208.84 | 12 | 12 | 121.62 | 7 | 7 | 83.75 | 3 | 3 |
| P40939 | Trifunctional enzyme subunit alpha mitochondrial GN=HADHA | 203.45 | 10 | 10 | 236.85 | 16 | 16 | 116.09 | 6 | 6 | 132.43 | 7 | 7 |
| P04406 | Glyceraldehyde-3-phosphate dehydrogenase GN=GAPDH | 226.38 | 10 | 10 | 210.97 | 10 | 10 | 111.39 | 3 | 3 | 78.53 | 2 | 2 |
| P62701 | 40S ribosomal protein S4 X isoform GN=RPS4X | 240.94 | 12 | 7 | 226.14 | 10 | 6 | 130.61 | 7 | 3 | 92.68 | 3 | 3 |
| P60709 | Actin cytoplasmic 1 GN=ACTB | 236.25 | 11 | 6 | 237.35 | 15 | 9 | 146.26 | 6 | 6 | 106.47 | 4 | 4 |
| P63261 | Actin cytoplasmic 2 GN=ACTG1 | 236.25 | 11 | 6 | 237.35 | 15 | 9 | 146.26 | 6 | 6 | 106.47 | 4 | 4 |
| Q9NVI7 | ATPase family AAA domain-containing protein 3A  GN=ATAD3A | 222.13 | 13 | 6 | 128.91 | 6 | 3 | 107.26 | 4 | 4 | 61.62 | 2 | 2 |
| Q9BQG0 | Myb-binding protein 1A GN=MYBBP1A | 133.74 | 5 | 5 | 307.43 | 30 | 30 | 80.25 | 4 | 4 | 88.45 | 4 | 4 |
| P06576 | ATP synthase subunit beta mitochondrial GN=ATP5F1B | 169.48 | 6 | 4 | 98.76 | 4 | 3 | 82.58 | 4 | 3 | 127.19 | 8 | 8 |
| P48047 | ATP synthase subunit O mitochondrial GN=ATP5PO | 145.58 | 4 | 4 | 135.5 | 4 | 4 | 67.36 | 2 | 2 | 79.03 | 2 | 2 |
| P05141 | ADP/ATP translocase 2 GN=SLC25A5 | 127.25 | 5 | 3 | 97.16 | 5 | 2 | 84.21 | 3 | 3 | 60.72 | 2 | 2 |
| Q00839 | Heterogeneous nuclear ribonucleoprotein U GN=HNRNPU | 239.09 | 16 | 16 | 253.46 | 21 | 21 | 129.88 | 8 | 8 | 41.74 | 1 | 1 |
| P02545 | Prelamin-A/C GN=LMNA | 187.98 | 15 | 14 | 249.39 | 21 | 20 | 73.03 | 3 | 3 | 44.05 | 1 | 1 |
| Q96PK6 | RNA-binding protein 14 GN=RBM14 | 232.17 | 13 | 13 | 194.91 | 12 | 12 | 141.42 | 9 | 9 | 39.37 | 1 | 1 |
| P61978 | Heterogeneous nuclear ribonucleoprotein K GN=HNRNPK | 224.32 | 12 | 12 | 196.34 | 10 | 10 | 94.69 | 4 | 4 | 37.67 | 1 | 1 |
| P33992 | DNA replication licensing factor MCM5 GN=MCM5 | 168.23 | 11 | 11 | 106.17 | 5 | 5 | 84.7 | 5 | 5 | 39.47 | 1 | 1 |
| P68363 | Tubulin alpha-1B chain GN=TUBA1B | 226.06 | 11 | 11 | 185.21 | 8 | 8 | 108.33 | 5 | 5 | 45.07 | 1 | 1 |
| O15240 | Neurosecretory protein VGF GN=VGF | 223.87 | 10 | 10 | 216.18 | 10 | 10 | 94.6 | 4 | 4 | 52.68 | 1 | 1 |
| P68104 | Elongation factor 1-alpha 1 GN=EEF1A1 | 160.53 | 9 | 9 | 111.19 | 6 | 6 | 94.57 | 3 | 3 | 38.43 | 1 | 1 |
| Q5VTE0 | Putative elongation factor 1-alpha-like 3 GN=EEF1A1P5 | 160.53 | 9 | 9 | 111.19 | 6 | 6 | 94.57 | 3 | 3 | 38.43 | 1 | 1 |
| Q9Y230 | RuvB-like 2 GN=RUVBL2 | 129.75 | 8 | 8 | 77.31 | 3 | 3 | 53.73 | 2 | 2 | 41.56 | 1 | 1 |
| Q9Y265 | RuvB-like 1 GN=RUVBL1 | 137.63 | 7 | 7 | 124.98 | 7 | 7 | 79.98 | 4 | 4 | 53.47 | 1 | 1 |
| Q13151 | Heterogeneous nuclear ribonucleoprotein A0 GN=HNRNPA0 | 174.12 | 6 | 6 | 140.85 | 5 | 5 | 97.03 | 3 | 3 | 43.95 | 1 | 1 |
| P06748 | Nucleophosmin GN=NPM1 | 189.66 | 6 | 6 | 151.94 | 6 | 6 | 92.27 | 3 | 3 | 36.78 | 1 | 1 |
| P62829 | 60S ribosomal protein L23 GN=RPL23 | 170.13 | 5 | 5 | 114.53 | 2 | 2 | 99.92 | 3 | 3 | 40.79 | 1 | 1 |
| P36542 | ATP synthase subunit gamma mitochondrial GN=ATP5F1C | 137.83 | 5 | 5 | 98.87 | 3 | 3 | 72.02 | 2 | 2 | 40.44 | 1 | 1 |
| Q14103 | Heterogeneous nuclear ribonucleoprotein D0 GN=HNRNPD | 135.58 | 5 | 4 | 124.82 | 3 | 3 | 88.75 | 3 | 3 | 41.47 | 1 | 1 |
| P22090 | 40S ribosomal protein S4 Y isoform 1 GN=RPS4Y1 | 186.02 | 8 | 3 | 180.21 | 7 | 3 | 115.15 | 6 | 2 | 40.5 | 1 | 1 |
| Q9Y2R4 | Probable ATP-dependent RNA helicase DDX52 GN=DDX52 | 171.51 | 7 | 7 | 260.36 | 14 | 14 | 31.17 | 1 | 1 | 39.47 | 1 | 1 |
| Q96CU9 | FAD-dependent oxidoreductase domain-containing protein 1 GN=FOXRED1 | 140.45 | 6 | 6 | 105.54 | 4 | 4 | 55.61 | 1 | 1 | 44.11 | 1 | 1 |
| Q9BVJ6 | U3 small nucleolar RNA-associated protein a14 homolog A GN=UTP14A | 115.79 | 6 | 6 | 176.73 | 11 | 11 | 40.68 | 1 | 1 | 37.01 | 1 | 1 |
| P11387 | DNA topoisomerase 1 GN=TOP1 | 104.88 | 5 | 5 | 109.39 | 4 | 4 | 36.39 | 1 | 1 | 35.94 | 1 | 1 |
| P60866 | 40S ribosomal protein S20 GN=RPS20 | 107.07 | 4 | 4 | 41.44 | 1 | 1 | 48.86 | 1 | 1 | 36.27 | 1 | 1 |
| P62266 | 40S ribosomal protein S23 GN=RPS23 | 126.67 | 4 | 4 | 81.34 | 3 | 3 | 43.51 | 1 | 1 | 36.51 | 1 | 1 |
| Q04837 | Single-stranded DNA-binding protein mitochondrial GN=SSBP1 | 128.23 | 3 | 3 | 123.44 | 4 | 4 | 30.9 | 1 | 1 | 46.64 | 1 | 1 |
| Q6P1L8 | 39S ribosomal protein L14 mitochondrial GN=MRPL14 | 93.62 | 2 | 2 | 47.37 | 1 | 1 | 36.71 | 1 | 1 | 43.59 | 1 | 1 |
| P02768 | Serum albumin GN=ALB | 98.57 | 2 | 2 | 96.29 | 3 | 3 | 41 | 1 | 1 | 74.4 | 2 | 2 |
| Q9H0D6 | 5'-3' exoribonuclease 2 GN=XRN2 | 183.01 | 14 | 14 | 181.25 | 10 | 10 | 91.05 | 3 | 3 |  |  |  |
| P25205 | DNA replication licensing factor MCM3 GN=MCM3 | 214.51 | 13 | 13 | 75.25 | 2 | 2 | 84.42 | 2 | 2 |  |  |  |
| Q6W2J9 | BCL-6 corepressor GN=BCOR | 153.73 | 12 | 12 | 249.45 | 20 | 20 | 64.64 | 2 | 2 |  |  |  |
| P22626 | Heterogeneous nuclear ribonucleoproteins A2/B1  GN=HNRNPA2B1 | 239.48 | 12 | 11 | 181.72 | 8 | 8 | 133.04 | 8 | 7 |  |  |  |
| O00148 | ATP-dependent RNA helicase DDX39A GN=DDX39A | 161.43 | 10 | 10 | 177.27 | 9 | 9 | 110.62 | 6 | 6 |  |  |  |
| Q13217 | DnaJ homolog subfamily C member 3 GN=DNAJC3 | 177.96 | 10 | 10 | 134.55 | 4 | 4 | 109.56 | 3 | 3 |  |  |  |
| P11021 | Endoplasmic reticulum chaperone BiP GN=HSPA5 | 198.45 | 12 | 10 | 97.58 | 6 | 5 | 76.46 | 4 | 3 |  |  |  |
| Q9Y446 | Plakophilin-3 GN=PKP3 | 167.55 | 9 | 9 | 56.11 | 2 | 2 | 89.97 | 4 | 4 |  |  |  |
| P26599 | Polypyrimidine tract-binding protein 1 GN=PTBP1 | 209.84 | 9 | 9 | 168.43 | 7 | 7 | 69.44 | 3 | 3 |  |  |  |
| P22087 | rRNA 2'-O-methyltransferase fibrillarin GN=FBL | 169.94 | 9 | 9 | 207.82 | 11 | 11 | 92.31 | 3 | 3 |  |  |  |
| Q9BY77 | Polymerase delta-interacting protein 3 GN=POLDIP3 | 193.19 | 8 | 8 | 168.57 | 5 | 5 | 103.06 | 4 | 4 |  |  |  |
| Q13148 | TAR DNA-binding protein 43 GN=TARDBP | 181.65 | 8 | 8 | 179.51 | 6 | 6 | 88.15 | 4 | 4 |  |  |  |
| P63244 | Receptor of activated protein C kinase 1 GN=RACK1 | 143.27 | 7 | 7 | 98.45 | 5 | 5 | 71.94 | 2 | 2 |  |  |  |
| P43243 | Matrin-3 GN=MATR3 | 145.43 | 6 | 6 | 172.71 | 12 | 12 | 80.33 | 3 | 3 |  |  |  |
| P09651 | Heterogeneous nuclear ribonucleoprotein A1 GN=HNRNPA1 | 177.44 | 7 | 6 | 129.26 | 4 | 4 | 102.55 | 3 | 2 |  |  |  |
| P22061 | Protein-L-isoaspartate(D-aspartate) O-methyltransferase GN=PCMT1 | 175.96 | 6 | 6 | 53.03 | 2 | 2 | 70.82 | 2 | 2 |  |  |  |
| O00571 | ATP-dependent RNA helicase DDX3X GN=DDX3X | 161.26 | 6 | 5 | 96.35 | 3 | 3 | 94.35 | 3 | 2 |  |  |  |
| P23528 | Cofilin-1 GN=CFL1 | 151.18 | 5 | 5 | 47.94 | 1 | 1 | 65.4 | 2 | 2 |  |  |  |
| Q92841 | Probable ATP-dependent RNA helicase DDX17 GN=DDX17 | 176.86 | 9 | 5 | 148.51 | 8 | 6 | 99.49 | 4 | 2 |  |  |  |
| Q15233 | Non-POU domain-containing octamer-binding protein  GN=NONO | 159.11 | 4 | 4 | 84.91 | 2 | 2 | 70.1 | 2 | 2 |  |  |  |
| Q14978 | Nucleolar and coiled-body phosphoprotein 1 GN=NOLC1 | 97.47 | 4 | 4 | 76.72 | 2 | 2 | 71.96 | 2 | 2 |  |  |  |
| P51648 | Aldehyde dehydrogenase family 3 member A2 GN=ALDH3A2 | 118.23 | 3 | 3 | 146.22 | 6 | 6 | 92.97 | 4 | 4 |  |  |  |
| P52597 | Heterogeneous nuclear ribonucleoprotein F  GN=HNRNPF | 169.03 | 4 | 3 | 139.47 | 3 | 2 | 93.36 | 3 | 2 |  |  |  |
| P68371 | Tubulin beta-4B chain GN=TUBB4B | 228.76 | 13 | 2 | 191.78 | 10 | 2 | 83.02 | 3 | 3 |  |  |  |
| Q9BSD7 | Cancer-related nucleoside-triphosphatase GN=NTPCR | 99.58 | 2 | 2 | 84.89 | 1 | 1 | 75.46 | 2 | 2 |  |  |  |
| P0DMV8 | Heat shock 70 kDa protein 1A GN=HSPA1A | 128.67 | 6 | 2 | 99.16 | 4 | 2 | 59.99 | 3 | 2 |  |  |  |
| P0DMV9 | Heat shock 70 kDa protein 1B GN=HSPA1B | 128.67 | 6 | 2 | 99.16 | 4 | 2 | 59.99 | 3 | 2 |  |  |  |
| P31943 | Heterogeneous nuclear ribonucleoprotein H GN=HNRNPH1 | 176.11 | 7 | 2 | 176.16 | 7 | 2 | 102.69 | 3 | 2 |  |  |  |
| P33993 | DNA replication licensing factor MCM7 GN=MCM7 | 191.09 | 14 | 14 | 167.84 | 10 | 10 | 36 | 1 | 1 |  |  |  |
| Q9NR30 | Nucleolar RNA helicase 2 GN=DDX21 | 140.64 | 10 | 10 | 159.84 | 9 | 9 | 46.85 | 1 | 1 |  |  |  |
| Q9UL03 | Integrator complex subunit 6 GN=INTS6 | 135.84 | 8 | 8 | 189.72 | 11 | 11 | 36.15 | 1 | 1 |  |  |  |
| Q96EY1 | DnaJ homolog subfamily A member 3 mitochondrial  GN=DNAJA3 | 180.72 | 7 | 7 | 129.51 | 6 | 6 | 44.54 | 1 | 1 |  |  |  |
| Q14938 | Nuclear factor 1 X-type GN=NFIX | 139.17 | 6 | 6 | 123.29 | 4 | 4 | 41.15 | 1 | 1 |  |  |  |
| P23246 | Splicing factor proline- and glutamine-rich GN=SFPQ | 180.74 | 6 | 6 | 85.7 | 4 | 4 | 60.05 | 1 | 1 |  |  |  |
| P49411 | Elongation factor Tu mitochondrial GN=TUFM | 171.22 | 5 | 5 | 104.23 | 3 | 3 | 38.49 | 1 | 1 |  |  |  |
| Q8NHM5 | Lysine-specific demethylase 2B GN=KDM2B | 127.33 | 5 | 5 | 91.2 | 5 | 5 | 46.02 | 1 | 1 |  |  |  |
| Q16795 | NADH dehydrogenase [ubiquinone] 1 alpha subcomplex subunit 9 mitochondrial GN=NDUFA9 | 108.58 | 5 | 5 | 78.39 | 3 | 3 | 35.41 | 1 | 1 |  |  |  |
| Q8NBN7 | Retinol dehydrogenase 13 GN=RDH13 | 113.15 | 5 | 5 | 112.88 | 4 | 4 | 41.25 | 1 | 1 |  |  |  |
| Q14498 | RNA-binding protein 39 GN=RBM39 | 112.81 | 5 | 5 | 161.73 | 6 | 6 | 31.26 | 1 | 1 |  |  |  |
| P31689 | DnaJ homolog subfamily A member 1 GN=DNAJA1 | 109.14 | 3 | 3 | 106.79 | 2 | 2 | 52.45 | 1 | 1 |  |  |  |
| Q9UHB6 | LIM domain and actin-binding protein 1 GN=LIMA1 | 70.72 | 3 | 3 | 110.79 | 5 | 5 | 34.83 | 1 | 1 |  |  |  |
| Q9Y285 | Phenylalanine--tRNA ligase alpha subunit GN=FARSA | 113.69 | 3 | 3 | 75.84 | 1 | 1 | 67.82 | 1 | 1 |  |  |  |
| Q9UJZ1 | Stomatin-like protein 2 mitochondrial GN=STOML2 | 136.14 | 3 | 3 | 60.93 | 1 | 1 | 45.1 | 1 | 1 |  |  |  |
| P12236 | ADP/ATP translocase 3 GN=SLC25A6 | 114.59 | 4 | 2 | 126.08 | 5 | 2 | 48.96 | 1 | 1 |  |  |  |
| P08574 | Cytochrome c1 heme protein mitochondrial GN=CYC1 | 53.65 | 2 | 2 | 72.75 | 2 | 2 | 31.99 | 1 | 1 |  |  |  |
| P04792 | Heat shock protein beta-1 GN=HSPB1 | 80.82 | 2 | 2 | 58.63 | 2 | 2 | 43.98 | 1 | 1 |  |  |  |
| Q00325 | Phosphate carrier protein mitochondrial GN=SLC25A3 | 61.34 | 2 | 2 | 55.19 | 1 | 1 | 35.72 | 1 | 1 |  |  |  |
| Q9UHV9 | Prefoldin subunit 2 GN=PFDN2 | 83.09 | 2 | 2 | 38.63 | 1 | 1 | 44.94 | 1 | 1 |  |  |  |
| Q15365 | Poly(rC)-binding protein 1 GN=PCBP1 | 145.74 | 5 | 3 | 75.01 | 2 | 1 | 32.97 | 1 | 0 |  |  |  |
| Q15366 | Poly(rC)-binding protein 2 GN=PCBP2 | 137.33 | 4 | 2 | 73.7 | 2 | 1 | 32.97 | 1 | 0 |  |  |  |
| Q8N163 | Cell cycle and apoptosis regulator protein 2 GN=CCAR2 | 30.9 | 1 | 1 | 221.85 | 15 | 15 | 47.92 | 2 | 2 |  |  |  |
| Q96BP2 | Coiled-coil-helix-coiled-coil-helix domain-containing protein 1 GN=CHCHD1 | 60.19 | 1 | 1 | 46.23 | 1 | 1 | 44.48 | 1 | 1 |  |  |  |
| P63167 | Dynein light chain 1 cytoplasmic GN=DYNLL1 | 73.62 | 1 | 1 | 94.77 | 1 | 1 | 31.44 | 1 | 1 |  |  |  |
| Q9NY12 | H/ACA ribonucleoprotein complex subunit 1 GN=GAR1 | 29.29 | 1 | 1 | 40.82 | 1 | 1 | 32.69 | 1 | 1 |  |  |  |
| P55795 | Heterogeneous nuclear ribonucleoprotein H2  GN=HNRNPH2 | 150.04 | 5 | 1 | 150.81 | 5 | 1 | 102.69 | 3 | 2 |  |  |  |
| P46013 | Proliferation marker protein Ki-67 GN=MKI67 | 48.11 | 1 | 1 | 169.12 | 13 | 13 | 35.56 | 1 | 1 |  |  |  |
| P32322 | Pyrroline-5-carboxylate reductase 1 mitochondrial  GN=PYCR1 | 55.96 | 2 | 1 | 95.85 | 3 | 2 | 57.24 | 2 | 2 |  |  |  |
| P63208 | S-phase kinase-associated protein 1 GN=SKP1 | 48.54 | 1 | 1 | 50.1 | 1 | 1 | 34.25 | 1 | 1 |  |  |  |
| P46783 | 40S ribosomal protein S10 GN=RPS10 | 92.13 | 3 | 3 | 77.3 | 2 | 2 | 72.29 | 2 | 2 |  |  |  |
| P62280 | 40S ribosomal protein S11 GN=RPS11 | 148.89 | 5 | 5 | 139.55 | 4 | 4 | 63.55 | 2 | 2 |  |  |  |
| P25398 | 40S ribosomal protein S12 GN=RPS12 | 76.51 | 2 | 2 | 77.27 | 2 | 2 | 39.64 | 1 | 1 |  |  |  |
| P62263 | 40S ribosomal protein S14 GN=RPS14 | 123.41 | 4 | 4 | 94.91 | 2 | 2 | 38.92 | 1 | 1 |  |  |  |
| P62841 | 40S ribosomal protein S15 GN=RPS15 | 136.95 | 4 | 4 | 97.54 | 3 | 3 | 64.73 | 2 | 2 |  |  |  |
| P08708 | 40S ribosomal protein S17 GN=RPS17 | 202.96 | 8 | 8 | 187.78 | 7 | 7 | 85.81 | 3 | 3 |  |  |  |
| P62269 | 40S ribosomal protein S18 GN=RPS18 | 138.13 | 6 | 6 | 120.78 | 7 | 7 | 58.37 | 2 | 2 |  |  |  |
| P62851 | 40S ribosomal protein S25 GN=RPS25 | 86.09 | 4 | 4 | 61.62 | 2 | 2 | 37.49 | 1 | 1 |  |  |  |
| P62857 | 40S ribosomal protein S28 GN=RPS28 | 46.18 | 1 | 1 | 68.75 | 1 | 1 | 40.46 | 1 | 1 |  |  |  |
| P61247 | 40S ribosomal protein S3a GN=RPS3A | 72.29 | 3 | 3 | 119.08 | 7 | 7 | 48.96 | 1 | 1 |  |  |  |
| P46782 | 40S ribosomal protein S5 GN=RPS5 | 170.58 | 4 | 4 | 157.99 | 5 | 5 | 76.77 | 2 | 2 |  |  |  |
| P62913 | 60S ribosomal protein L11 GN=RPL11 | 113.87 | 3 | 3 | 92.95 | 2 | 2 | 62.57 | 2 | 2 |  |  |  |
| P26373 | 60S ribosomal protein L13 GN=RPL13 | 66.4 | 2 | 2 | 93.8 | 4 | 4 | 33.31 | 1 | 1 |  |  |  |
| Q07020 | 60S ribosomal protein L18 GN=RPL18 | 37.58 | 1 | 1 | 49.04 | 2 | 2 | 35.68 | 1 | 1 |  |  |  |
| Q6P5R6 | 60S ribosomal protein L22-like 1 GN=RPL22L1 | 89.03 | 3 | 3 | 140.51 | 3 | 3 | 68.71 | 2 | 2 |  |  |  |
| P32969 | 60S ribosomal protein L9 GN=RPL9 | 158.85 | 5 | 5 | 126.35 | 4 | 4 | 31.11 | 1 | 1 |  |  |  |
| Q9NUL7 | Probable ATP-dependent RNA helicase DDX28 GN=DDX28 | 178.73 | 9 | 9 | 68.45 | 2 | 2 |  |  |  | 50.68 | 1 | 1 |
| P12004 | Proliferating cell nuclear antigen GN=PCNA | 146.66 | 5 | 5 | 101.89 | 2 | 2 |  |  |  | 47.28 | 1 | 1 |
| P05388 | 60S acidic ribosomal protein P0 GN=RPLP0 | 124.39 | 4 | 4 | 33.04 | 1 | 1 |  |  |  | 48.54 | 1 | 1 |
| Q8NHW5 | 60S acidic ribosomal protein P0-like GN=RPLP0P6 | 124.39 | 4 | 4 | 33.04 | 1 | 1 |  |  |  | 48.54 | 1 | 1 |
| P07437 | Tubulin beta chain GN=TUBB | 224.36 | 13 | 3 | 183.92 | 9 | 1 |  |  |  | 37.2 | 1 | 1 |
| P55084 | Trifunctional enzyme subunit beta mitochondrial  GN=HADHB | 58.91 | 2 | 2 | 87.35 | 4 | 4 |  |  |  | 93.44 | 3 | 3 |
| Q9H7Z7 | Prostaglandin E synthase 2 GN=PTGES2 | 72.52 | 2 | 2 | 146.03 | 5 | 5 |  |  |  | 44.59 | 1 | 1 |
| O43819 | Protein SCO2 homolog mitochondrial GN=SCO2 | 90.56 | 1 | 1 | 74.79 | 1 | 1 |  |  |  | 38.11 | 1 | 1 |
| Q12905 | Interleukin enhancer-binding factor 2 GN=ILF2 | 41.81 | 1 | 1 | 142.93 | 5 | 5 |  |  |  | 43.86 | 1 | 1 |
| Q8NBQ5 | Estradiol 17-beta-dehydrogenase 11 GN=HSD17B11 | 29.32 | 1 | 1 | 117.32 | 2 | 2 |  |  |  | 67.04 | 2 | 2 |
| P62879 | Guanine nucleotide-binding protein G(I)/G(S)/G(T) subunit beta-2 GN=GNB2 | 33.74 | 1 | 1 | 138.84 | 5 | 2 |  |  |  | 53.45 | 1 | 1 |
| P62873 | Guanine nucleotide-binding protein G(I)/G(S)/G(T) subunit beta-1 GN=GNB1 | 33.74 | 1 | 1 | 107.83 | 4 | 1 |  |  |  | 37.58 | 1 | 1 |
| Q99729 | Heterogeneous nuclear ribonucleoprotein A/B  GN=HNRNPAB | 51.01 | 2 | 1 | 66.68 | 1 | 1 |  |  |  | 41.47 | 1 | 1 |
| Q86YZ3 | Hornerin GN=HRNR | 114.61 | 4 | 4 |  |  |  | 123.67 | 4 | 4 | 67.48 | 2 | 2 |
| P05387 | 60S acidic ribosomal protein P2 GN=RPLP2 | 55.1 | 1 | 1 |  |  |  | 31.92 | 1 | 1 | 43.84 | 1 | 1 |
| A1L0T0 | Acetolactate synthase-like protein GN=ILVBL |  |  |  | 115.64 | 2 | 2 | 37.94 | 1 | 1 |  |  |  |
| Q15599 | Na(+)/H(+) exchange regulatory cofactor NHE-RF2  GN=SLC9A3R2 | 165.9 | 7 | 7 | 91.71 | 2 | 2 |  |  |  |  |  |  |
| O15119 | T-box transcription factor TBX3 GN=TBX3 | 167.21 | 6 | 6 | 144.19 | 5 | 5 |  |  |  |  |  |  |
| P22830 | Ferrochelatase mitochondrial GN=FECH | 92.75 | 4 | 4 | 111.05 | 4 | 4 |  |  |  |  |  |  |
| P55265 | Double-stranded RNA-specific adenosine deaminase  GN=ADAR | 95.37 | 3 | 3 | 210.6 | 15 | 15 |  |  |  |  |  |  |
| O75643 | U5 small nuclear ribonucleoprotein 200 kDa helicase  GN=SNRNP200 | 99.28 | 3 | 3 | 161.89 | 10 | 10 |  |  |  |  |  |  |
| P42167 | Lamina-associated polypeptide 2 isoforms beta/gamma  GN=TMPO | 91.63 | 3 | 3 | 249.3 | 13 | 7 |  |  |  |  |  |  |
| P09543 | 2' 3'-cyclic-nucleotide 3'-phosphodiesterase GN=CNP | 92.83 | 3 | 3 | 155.84 | 5 | 5 |  |  |  |  |  |  |
| Q99496 | E3 ubiquitin-protein ligase RING2 GN=RNF2 | 84.45 | 3 | 3 | 160.17 | 6 | 4 |  |  |  |  |  |  |
| P14866 | Heterogeneous nuclear ribonucleoprotein L GN=HNRNPL | 78.7 | 3 | 3 | 88.51 | 4 | 4 |  |  |  |  |  |  |
| P63173 | 60S ribosomal protein L38 GN=RPL38 | 89.28 | 3 | 3 | 84.94 | 3 | 3 |  |  |  |  |  |  |
| Q07666 | KH domain-containing RNA-binding signal transduction associated protein 1 GN=KHDRBS1 | 71.48 | 3 | 3 | 81.5 | 3 | 3 |  |  |  |  |  |  |
| Q9NX20 | 39S ribosomal protein L16 mitochondrial GN=MRPL16 | 74.14 | 3 | 3 | 77.41 | 2 | 2 |  |  |  |  |  |  |
| P08047 | Transcription factor Sp1 GN=SP1 | 117.36 | 3 | 3 | 92.32 | 2 | 2 |  |  |  |  |  |  |
| P20700 | Lamin-B1 GN=LMNB1 | 91.53 | 3 | 2 | 168.18 | 13 | 12 |  |  |  |  |  |  |
| O00567 | Nucleolar protein 56 GN=NOP56 | 64.22 | 2 | 2 | 185.89 | 9 | 9 |  |  |  |  |  |  |
| Q9Y2X3 | Nucleolar protein 58 GN=NOP58 | 69.71 | 2 | 2 | 162.75 | 9 | 9 |  |  |  |  |  |  |
| Q9H0A0 | RNA cytidine acetyltransferase GN=NAT10 | 87.1 | 2 | 2 | 156.4 | 7 | 7 |  |  |  |  |  |  |
| P36873 | Serine/threonine-protein phosphatase PP1-gamma catalytic subunit GN=PPP1CC | 58.97 | 2 | 2 | 126.02 | 6 | 6 |  |  |  |  |  |  |
| P23769 | Endothelial transcription factor GATA2 GN=GATA2 | 91.14 | 2 | 2 | 168.34 | 5 | 5 |  |  |  |  |  |  |
| Q8IY67 | Ribonucleoprotein PTB-binding 1 GN=RAVER1 | 61.67 | 2 | 2 | 119.32 | 4 | 4 |  |  |  |  |  |  |
| Q9BSM1 | Polycomb group RING finger protein 1 GN=PCGF1 | 48.21 | 2 | 2 | 111.12 | 3 | 3 |  |  |  |  |  |  |
| P84090 | Enhancer of rudimentary homolog GN=ERH | 104.19 | 2 | 2 | 103.91 | 2 | 2 |  |  |  |  |  |  |
| Q96EE3 | Nucleoporin SEH1 GN=SEH1L | 80.69 | 2 | 2 | 99.07 | 2 | 2 |  |  |  |  |  |  |
| P62318 | Small nuclear ribonucleoprotein Sm D3 GN=SNRPD3 | 67.62 | 2 | 2 | 66.91 | 2 | 2 |  |  |  |  |  |  |
| Q5T9A4 | ATPase family AAA domain-containing protein 3B  GN=ATAD3B | 153.2 | 8 | 1 | 74.69 | 4 | 1 |  |  |  |  |  |  |
| P68032 | Actin alpha cardiac muscle 1 GN=ACTC1 | 177.01 | 6 | 1 | 165.91 | 7 | 1 |  |  |  |  |  |  |
| P68133 | Actin alpha skeletal muscle GN=ACTA1 | 177.01 | 6 | 1 | 165.91 | 7 | 1 |  |  |  |  |  |  |
| P62736 | Actin aortic smooth muscle GN=ACTA2 | 177.01 | 6 | 1 | 165.91 | 7 | 1 |  |  |  |  |  |  |
| P63267 | Actin gamma-enteric smooth muscle GN=ACTG2 | 177.01 | 6 | 1 | 165.91 | 7 | 1 |  |  |  |  |  |  |
| Q8IWS0 | PHD finger protein 6 GN=PHF6 | 99.58 | 5 | 5 | 43.39 | 1 | 1 |  |  |  |  |  |  |
| P62249 | 40S ribosomal protein S16 GN=RPS16 | 99.02 | 4 | 4 | 29.34 | 1 | 1 |  |  |  |  |  |  |
| Q9NUI1 | Peroxisomal 2 4-dienoyl-CoA reductase GN=DECR2 | 84.47 | 4 | 4 | 30.2 | 1 | 1 |  |  |  |  |  |  |
| P61254 | 60S ribosomal protein L26 GN=RPL26 | 72.94 | 3 | 3 | 29.13 | 1 | 1 |  |  |  |  |  |  |
| O43390 | Heterogeneous nuclear ribonucleoprotein R GN=HNRNPR | 97.68 | 3 | 2 | 74.8 | 2 | 1 |  |  |  |  |  |  |
| P18085 | ADP-ribosylation factor 4 GN=ARF4 | 66.89 | 2 | 2 | 55.09 | 1 | 1 |  |  |  |  |  |  |
| P56134 | ATP synthase subunit f mitochondrial GN=ATP5MF | 70.84 | 2 | 2 | 29.88 | 1 | 1 |  |  |  |  |  |  |
| P13995 | Bifunctional methylenetetrahydrofolate dehydrogenase/cyclohydrolase mitochondrial GN=MTHFD2 | 60.41 | 2 | 2 | 28.52 | 1 | 1 |  |  |  |  |  |  |
| Q96EP5 | DAZ-associated protein 1 GN=DAZAP1 | 84.85 | 2 | 2 | 41.72 | 1 | 1 |  |  |  |  |  |  |
| Q9NV06 | DDB1- and CUL4-associated factor 13 GN=DCAF13 | 48.87 | 2 | 2 | 61.9 | 1 | 1 |  |  |  |  |  |  |
| O60884 | DnaJ homolog subfamily A member 2 GN=DNAJA2 | 68.6 | 2 | 2 | 67.8 | 1 | 1 |  |  |  |  |  |  |
| P26641 | Elongation factor 1-gamma GN=EEF1G | 48.5 | 2 | 2 | 30.71 | 1 | 1 |  |  |  |  |  |  |
| O95900 | Mitochondrial mRNA pseudouridine synthase TRUB2  GN=TRUB2 | 51.76 | 2 | 2 | 35.53 | 1 | 1 |  |  |  |  |  |  |
| P37108 | Signal recognition particle 14 kDa protein GN=SRP14 | 91.44 | 2 | 2 | 47.76 | 1 | 1 |  |  |  |  |  |  |
| Q9Y2W1 | Thyroid hormone receptor-associated protein 3  GN=THRAP3 | 56.82 | 2 | 2 | 40.37 | 1 | 1 |  |  |  |  |  |  |
| O60506 | Heterogeneous nuclear ribonucleoprotein Q  GN=SYNCRIP | 72.44 | 2 | 1 | 53.97 | 2 | 1 |  |  |  |  |  |  |
| Q15029 | 116 kDa U5 small nuclear ribonucleoprotein component  GN=EFTUD2 | 64.21 | 1 | 1 | 129.79 | 5 | 5 |  |  |  |  |  |  |
| P51153 | Ras-related protein Rab-13 GN=RAB13 | 40.44 | 1 | 1 | 150.02 | 5 | 4 |  |  |  |  |  |  |
| Q13724 | Mannosyl-oligosaccharide glucosidase GN=MOGS | 53.5 | 1 | 1 | 141.01 | 4 | 4 |  |  |  |  |  |  |
| Q14527 | Helicase-like transcription factor GN=HLTF | 29.04 | 1 | 1 | 98.54 | 3 | 3 |  |  |  |  |  |  |
| Q96C36 | Pyrroline-5-carboxylate reductase 2 GN=PYCR2 | 38.49 | 1 | 0 | 80.74 | 3 | 2 |  |  |  |  |  |  |
| P62753 | 40S ribosomal protein S6 GN=RPS6 | 50.36 | 1 | 1 | 66.42 | 2 | 2 |  |  |  |  |  |  |
| Q13547 | Histone deacetylase 1 GN=HDAC1 | 29.9 | 1 | 1 | 67.38 | 2 | 2 |  |  |  |  |  |  |
| Q5C9Z4 | Nucleolar MIF4G domain-containing protein 1 GN=NOM1 | 47.92 | 1 | 1 | 71.9 | 2 | 2 |  |  |  |  |  |  |
| Q8IY37 | Probable ATP-dependent RNA helicase DHX37  GN=DHX37 | 46.74 | 1 | 1 | 51.32 | 2 | 2 |  |  |  |  |  |  |
| O43824 | Putative GTP-binding protein 6 GN=GTPBP6 | 73.87 | 1 | 1 | 49.27 | 2 | 2 |  |  |  |  |  |  |
| Q15637 | Splicing factor 1 GN=SF1 | 37.97 | 1 | 1 | 52.39 | 2 | 2 |  |  |  |  |  |  |
| P82930 | 28S ribosomal protein S34 mitochondrial GN=MRPS34 | 58.32 | 1 | 1 | 39.18 | 1 | 1 |  |  |  |  |  |  |
| Q9NVE7 | 4'-phosphopantetheine phosphatase GN=PANK4 | 33.4 | 1 | 1 | 30.62 | 1 | 1 |  |  |  |  |  |  |
| P40429 | 60S ribosomal protein L13a GN=RPL13A | 29.55 | 1 | 1 | 30.37 | 1 | 1 |  |  |  |  |  |  |
| P50914 | 60S ribosomal protein L14 GN=RPL14 | 39.68 | 1 | 1 | 30.96 | 1 | 1 |  |  |  |  |  |  |
| P61353 | 60S ribosomal protein L27 GN=RPL27 | 32.76 | 1 | 1 | 45.53 | 1 | 1 |  |  |  |  |  |  |
| P62910 | 60S ribosomal protein L32 GN=RPL32 | 63.74 | 1 | 1 | 68.82 | 1 | 1 |  |  |  |  |  |  |
| P61513 | 60S ribosomal protein L37a GN=RPL37A | 62.34 | 1 | 1 | 28.48 | 1 | 1 |  |  |  |  |  |  |
| O76031 | ATP-dependent Clp protease ATP-binding subunit clpX like mitochondrial GN=CLPX | 34.99 | 1 | 1 | 38.27 | 1 | 1 |  |  |  |  |  |  |
| Q9Y3Y2 | Chromatin target of PRMT1 protein GN=CHTOP | 51.46 | 1 | 1 | 48.88 | 1 | 1 |  |  |  |  |  |  |
| Q07021 | Complement component 1 Q subcomponent-binding protein mitochondrial GN=C1QBP | 54.53 | 1 | 1 | 32.47 | 1 | 1 |  |  |  |  |  |  |
| Q13363 | C-terminal-binding protein 1 GN=CTBP1 | 34.87 | 1 | 1 | 40.06 | 1 | 1 |  |  |  |  |  |  |
| O60762 | Dolichol-phosphate mannosyltransferase subunit 1  GN=DPM1 | 43.36 | 1 | 1 | 48.56 | 1 | 1 |  |  |  |  |  |  |
| O60832 | H/ACA ribonucleoprotein complex subunit DKC1  GN=DKC1 | 48.55 | 1 | 1 | 40.62 | 1 | 1 |  |  |  |  |  |  |
| P16401 | Histone H1.5 GN=H1-5 | 32.37 | 1 | 1 | 31.91 | 1 | 1 |  |  |  |  |  |  |
| P33778 | Histone H2B type 1-B GN=HIST1H2BB | 47.62 | 1 | 1 | 43.61 | 1 | 1 |  |  |  |  |  |  |
| P62807 | Histone H2B type 1-C/E/F/G/I GN=H2BC4 | 47.62 | 1 | 1 | 43.61 | 1 | 1 |  |  |  |  |  |  |
| P58876 | Histone H2B type 1-D GN=HIST1H2BD | 47.62 | 1 | 1 | 43.61 | 1 | 1 |  |  |  |  |  |  |
| Q93079 | Histone H2B type 1-H GN=HIST1H2BH | 47.62 | 1 | 1 | 43.61 | 1 | 1 |  |  |  |  |  |  |
| P06899 | Histone H2B type 1-J GN=H2BC11 | 47.62 | 1 | 1 | 43.61 | 1 | 1 |  |  |  |  |  |  |
| O60814 | Histone H2B type 1-K GN=H2BC12 | 47.62 | 1 | 1 | 43.61 | 1 | 1 |  |  |  |  |  |  |
| Q99880 | Histone H2B type 1-L GN=H2BC13 | 47.62 | 1 | 1 | 43.61 | 1 | 1 |  |  |  |  |  |  |
| Q99879 | Histone H2B type 1-M GN=H2BC14 | 47.62 | 1 | 1 | 43.61 | 1 | 1 |  |  |  |  |  |  |
| Q99877 | Histone H2B type 1-N GN=H2BC15 | 47.62 | 1 | 1 | 43.61 | 1 | 1 |  |  |  |  |  |  |
| P23527 | Histone H2B type 1-O GN=HIST1H2BO | 47.62 | 1 | 1 | 43.61 | 1 | 1 |  |  |  |  |  |  |
| Q16778 | Histone H2B type 2-E GN=HIST2H2BE | 47.62 | 1 | 1 | 43.61 | 1 | 1 |  |  |  |  |  |  |
| Q5QNW6 | Histone H2B type 2-F GN=HIST2H2BF | 47.62 | 1 | 1 | 43.61 | 1 | 1 |  |  |  |  |  |  |
| Q8N257 | Histone H2B type 3-B GN=HIST3H2BB | 47.62 | 1 | 1 | 43.61 | 1 | 1 |  |  |  |  |  |  |
| P57053 | Histone H2B type F-S GN=H2BFS | 47.62 | 1 | 1 | 43.61 | 1 | 1 |  |  |  |  |  |  |
| Q3ZCT8 | Kelch repeat and BTB domain-containing protein 12  GN=KBTBD12 | 31.75 | 1 | 1 | 33.06 | 1 | 1 |  |  |  |  |  |  |
| P56270 | Myc-associated zinc finger protein GN=MAZ | 44.2 | 1 | 1 | 41.57 | 1 | 1 |  |  |  |  |  |  |
| Q8WUK0 | Phosphatidylglycerophosphatase and protein-tyrosine phosphatase 1 GN=PTPMT1 | 50.24 | 1 | 1 | 39.87 | 1 | 1 |  |  |  |  |  |  |
| Q53GA4 | Pleckstrin homology-like domain family A member 2  GN=PHLDA2 | 50.76 | 1 | 1 | 46.48 | 1 | 1 |  |  |  |  |  |  |
| P61619 | Protein transport protein Sec61 subunit alpha isoform 1  GN=SEC61A1 | 39.64 | 1 | 1 | 43.06 | 1 | 1 |  |  |  |  |  |  |
| Q6NVV1 | Putative 60S ribosomal protein L13a protein RPL13AP3  GN=RPL13AP3 | 29.55 | 1 | 1 | 30.37 | 1 | 1 |  |  |  |  |  |  |
| P35250 | Replication factor C subunit 2 GN=RFC2 | 46.85 | 1 | 1 | 57.72 | 1 | 1 |  |  |  |  |  |  |
| Q96T37 | RNA-binding protein 15 GN=RBM15 | 45.44 | 1 | 1 | 41.7 | 1 | 1 |  |  |  |  |  |  |
| P62306 | Small nuclear ribonucleoprotein F GN=SNRPF | 60.17 | 1 | 1 | 54.12 | 1 | 1 |  |  |  |  |  |  |
| P62314 | Small nuclear ribonucleoprotein Sm D1 GN=SNRPD1 | 71.6 | 1 | 1 | 59.1 | 1 | 1 |  |  |  |  |  |  |
| Q7Z2T5 | TRMT1-like protein GN=TRMT1L | 50.61 | 1 | 1 | 78.97 | 1 | 1 |  |  |  |  |  |  |
| P15880 | 40S ribosomal protein S2 GN=RPS2 | 84.02 | 5 | 5 | 90.68 | 3 | 3 |  |  |  |  |  |  |
| P62277 | 40S ribosomal protein S13 GN=RPS13 | 116.84 | 4 | 4 | 99.64 | 3 | 3 |  |  |  |  |  |  |
| P62244 | 40S ribosomal protein S15a GN=RPS15A | 82.04 | 2 | 2 | 75.56 | 2 | 2 |  |  |  |  |  |  |
| P39019 | 40S ribosomal protein S19 GN=RPS19 | 72.85 | 2 | 2 | 66.43 | 2 | 2 |  |  |  |  |  |  |
| P62273 | 40S ribosomal protein S29 GN=RPS29 | 54.23 | 2 | 2 | 58.9 | 2 | 2 |  |  |  |  |  |  |
| P62081 | 40S ribosomal protein S7 GN=RPS7 | 66.75 | 2 | 2 | 65.3 | 2 | 2 |  |  |  |  |  |  |
| P27635 | 60S ribosomal protein L10 GN=RPL10 | 81.57 | 2 | 2 | 85.52 | 3 | 3 |  |  |  |  |  |  |
| P18621 | 60S ribosomal protein L17 GN=RPL17 | 60.61 | 2 | 2 | 83.81 | 2 | 2 |  |  |  |  |  |  |
| P46778 | 60S ribosomal protein L21 GN=RPL21 | 75.33 | 2 | 2 | 51.07 | 1 | 1 |  |  |  |  |  |  |
| P83731 | 60S ribosomal protein L24 GN=RPL24 | 82.92 | 2 | 2 | 33.01 | 1 | 1 |  |  |  |  |  |  |
| P46776 | 60S ribosomal protein L27a GN=RPL27A | 49.57 | 2 | 2 | 39.48 | 1 | 1 |  |  |  |  |  |  |
| Q86V81 | THO complex subunit 4 GN=ALYREF | 212.79 | 8 | 8 |  |  |  | 61.45 | 2 | 2 |  |  |  |
| O95831 | Apoptosis-inducing factor 1 mitochondrial GN=AIFM1 | 125.16 | 5 | 5 |  |  |  | 53.86 | 2 | 2 |  |  |  |
| P08238 | Heat shock protein HSP 90-beta GN=HSP90AB1 | 132.58 | 5 | 5 |  |  |  | 53.38 | 2 | 2 |  |  |  |
| P78347 | General transcription factor II-I GN=GTF2I | 104.94 | 4 | 4 |  |  |  | 57.92 | 2 | 2 |  |  |  |
| P35637 | RNA-binding protein FUS GN=FUS | 74.33 | 3 | 3 |  |  |  | 58.84 | 2 | 2 |  |  |  |
| P42704 | Leucine-rich PPR motif-containing protein mitochondrial  GN=LRPPRC | 83.43 | 2 | 2 |  |  |  | 39.48 | 1 | 1 |  |  |  |
| O75489 | NADH dehydrogenase [ubiquinone] iron-sulphur protein 3 mitochondrial GN=NDUFS3 | 58.6 | 2 | 2 |  |  |  | 33.62 | 1 | 1 |  |  |  |
| Q9Y4W6 | AFG3-like protein 2 GN=AFG3L2 | 37 | 1 | 1 |  |  |  | 34.16 | 1 | 1 |  |  |  |
| Q02978 | Mitochondrial 2-oxoglutarate/malate carrier protein  GN=SLC25A11 | 34.67 | 1 | 1 |  |  |  | 38.08 | 1 | 1 |  |  |  |
| Q9H936 | Mitochondrial glutamate carrier 1 GN=SLC25A22 | 33.13 | 1 | 1 |  |  |  | 40.36 | 1 | 1 |  |  |  |
| Q9H1K4 | Mitochondrial glutamate carrier 2 GN=SLC25A18 | 33.13 | 1 | 1 |  |  |  | 40.36 | 1 | 1 |  |  |  |
| Q15436 | Protein transport protein Sec23A GN=SEC23A | 45.62 | 1 | 1 |  |  |  | 43.48 | 1 | 1 |  |  |  |
| Q15437 | Protein transport protein Sec23B GN=SEC23B | 45.62 | 1 | 1 |  |  |  | 43.48 | 1 | 1 |  |  |  |
| Q9GZT3 | SRA stem-loop-interacting RNA-binding protein mitochondrial GN=SLIRP | 40.26 | 1 | 1 |  |  |  | 36.94 | 1 | 1 |  |  |  |
| P53007 | Tricarboxylate transport protein mitochondrial  GN=SLC25A1 | 31.07 | 1 | 1 |  |  |  | 36.26 | 1 | 1 |  |  |  |
| P07477 | Trypsin-1 GN=PRSS1 | 90.83 | 2 | 2 |  |  |  |  |  |  | 51.31 | 1 | 1 |
| O00483 | Cytochrome c oxidase subunit NDUFA4 GN=NDUFA4 |  |  |  | 34.38 | 1 | 1 | 45.98 | 1 | 1 |  |  |  |
| P24539 | ATP synthase F(0) complex subunit B1 mitochondrial  GN=ATP5PB |  |  |  | 71.65 | 2 | 2 | 48.66 | 1 | 1 |  |  |  |
| Q15072 | Zinc finger protein OZF GN=ZNF146 |  |  |  | 87.14 | 3 | 3 | 52.02 | 1 | 1 |  |  |  |
| Q9BQE3 | Tubulin alpha-1C chain GN=TUBA1C |  |  |  | 185.21 | 8 | 8 |  |  |  | 45.07 | 1 | 1 |
| P11233 | Ras-related protein Ral-A GN=RALA |  |  |  | 138.92 | 4 | 4 |  |  |  | 43.35 | 1 | 1 |
| P61224 | Ras-related protein Rap-1b GN=RAP1B |  |  |  | 89.78 | 3 | 3 |  |  |  | 41.45 | 1 | 1 |
| P27824 | Calnexin GN=CANX |  |  |  | 80.86 | 2 | 2 |  |  |  | 37.11 | 1 | 1 |
| Q86UE4 | Protein LYRIC GN=MTDH |  |  |  | 39.11 | 1 | 1 |  |  |  | 35.77 | 1 | 1 |
| P51149 | Ras-related protein Rab-7a GN=RAB7A |  |  |  | 29.04 | 1 | 1 |  |  |  | 49.49 | 1 | 1 |
| P22695 | Cytochrome b-c1 complex subunit 2 mitochondrial  GN=UQCRC2 |  |  |  | 60.07 | 1 | 1 |  |  |  | 58.95 | 1 | 1 |
| O94826 | Mitochondrial import receptor subunit TOM70  GN=TOMM70 |  |  |  | 28.83 | 1 | 1 |  |  |  | 49.54 | 1 | 1 |
| Q5D862 | Filaggrin-2 GN=FLG2 |  |  |  |  |  |  | 41.94 | 1 | 1 | 42.08 | 1 | 1 |
| Q86U44 | N6-adenosine-methyltransferase catalytic subunit  GN=METTL3 | 132.03 | 4 | 4 |  |  |  |  |  |  |  |  |  |
| Q9Y2R9 | 28S ribosomal protein S7 mitochondrial GN=MRPS7 | 98.36 | 3 | 3 |  |  |  |  |  |  |  |  |  |
| P62805 | Histone H4 GN=H4C1 | 83.97 | 3 | 3 |  |  |  |  |  |  |  |  |  |
| P67809 | Y-box-binding protein 1 GN=YBX1 | 130.23 | 3 | 3 |  |  |  |  |  |  |  |  |  |
| P08195 | 4F2 cell-surface antigen heavy chain GN=SLC3A2 | 63.27 | 2 | 2 |  |  |  |  |  |  |  |  |  |
| P62899 | 60S ribosomal protein L31 GN=RPL31 | 47.28 | 2 | 2 |  |  |  |  |  |  |  |  |  |
| Q9NXV6 | CDKN2A-interacting protein GN=CDKN2AIP | 63.3 | 2 | 2 |  |  |  |  |  |  |  |  |  |
| P60981 | Destrin GN=DSTN | 52.73 | 2 | 2 |  |  |  |  |  |  |  |  |  |
| Q92945 | Far upstream element-binding protein 2 GN=KHSRP | 57.11 | 2 | 2 |  |  |  |  |  |  |  |  |  |
| P68871 | Haemoglobin subunit beta GN=HBB | 68.86 | 2 | 2 |  |  |  |  |  |  |  |  |  |
| Q92615 | La-related protein 4B GN=LARP4B | 52.61 | 2 | 2 |  |  |  |  |  |  |  |  |  |
| Q01650 | Large neutral amino acids transporter small subunit 1  GN=SLC7A5 | 76.71 | 2 | 2 |  |  |  |  |  |  |  |  |  |
| P29966 | Myristoylated alanine-rich C-kinase substrate GN=MARCKS | 74.64 | 2 | 2 |  |  |  |  |  |  |  |  |  |
| Q9P258 | Protein RCC2 GN=RCC2 | 53.5 | 2 | 2 |  |  |  |  |  |  |  |  |  |
| P62136 | Serine/threonine-protein phosphatase PP1-alpha catalytic subunit GN=PPP1CA | 58.97 | 2 | 2 |  |  |  |  |  |  |  |  |  |
| P62140 | Serine/threonine-protein phosphatase PP1-beta catalytic subunit GN=PPP1CB | 58.97 | 2 | 2 |  |  |  |  |  |  |  |  |  |
| P82914 | 28S ribosomal protein S15 mitochondrial GN=MRPS15 | 31.07 | 1 | 1 |  |  |  |  |  |  |  |  |  |
| Q9Y399 | 28S ribosomal protein S2 mitochondrial GN=MRPS2 | 37.19 | 1 | 1 |  |  |  |  |  |  |  |  |  |
| Q9Y2Q9 | 28S ribosomal protein S28 mitochondrial GN=MRPS28 | 30.69 | 1 | 1 |  |  |  |  |  |  |  |  |  |
| P42677 | 40S ribosomal protein S27 GN=RPS27 | 39.12 | 1 | 1 |  |  |  |  |  |  |  |  |  |
| P62241 | 40S ribosomal protein S8 GN=RPS8 | 38.47 | 1 | 1 |  |  |  |  |  |  |  |  |  |
| P08865 | 40S ribosomal protein SA GN=RPSA | 29.36 | 1 | 1 |  |  |  |  |  |  |  |  |  |
| P30050 | 60S ribosomal protein L12 GN=RPL12 | 36.94 | 1 | 1 |  |  |  |  |  |  |  |  |  |
| P61313 | 60S ribosomal protein L15 GN=RPL15 | 56.85 | 1 | 1 |  |  |  |  |  |  |  |  |  |
| P84098 | 60S ribosomal protein L19 GN=RPL19 | 49.04 | 1 | 1 |  |  |  |  |  |  |  |  |  |
| P46779 | 60S ribosomal protein L28 GN=RPL28 | 34.32 | 1 | 1 |  |  |  |  |  |  |  |  |  |
| P47914 | 60S ribosomal protein L29 GN=RPL29 | 48.15 | 1 | 1 |  |  |  |  |  |  |  |  |  |
| P83881 | 60S ribosomal protein L36a GN=RPL36A | 38.33 | 1 | 1 |  |  |  |  |  |  |  |  |  |
| Q969Q0 | 60S ribosomal protein L36a-like GN=RPL36AL | 38.33 | 1 | 1 |  |  |  |  |  |  |  |  |  |
| P62424 | 60S ribosomal protein L7a GN=RPL7A | 35.68 | 1 | 1 |  |  |  |  |  |  |  |  |  |
| Q9H0C2 | ADP/ATP translocase 4 GN=SLC25A31 | 51.23 | 2 | 1 |  |  |  |  |  |  |  |  |  |
| Q9NXU5 | ADP-ribosylation factor-like protein 15 GN=ARL15 | 30.06 | 1 | 1 |  |  |  |  |  |  |  |  |  |
| O00116 | Alkyl dihydroxyacetone phosphate synthase peroxisomal GN=AGPS | 44.49 | 1 | 1 |  |  |  |  |  |  |  |  |  |
| Q9NUU7 | ATP-dependent RNA helicase DDX19A GN=DDX19A | 34.74 | 1 | 1 |  |  |  |  |  |  |  |  |  |
| Q9UMR2 | ATP-dependent RNA helicase DDX19B GN=DDX19B | 34.74 | 1 | 1 |  |  |  |  |  |  |  |  |  |
| Q96SN8 | CDK5 regulatory subunit-associated protein 2  GN=CDK5RAP2 | 28.95 | 1 | 1 |  |  |  |  |  |  |  |  |  |
| Q96AJ1 | Clusterin-associated protein 1 GN=CLUAP1 | 37.75 | 1 | 1 |  |  |  |  |  |  |  |  |  |
| Q9H845 | Complex I assembly factor ACAD9 mitochondrial  GN=ACAD9 | 69.49 | 1 | 1 |  |  |  |  |  |  |  |  |  |
| Q02338 | D-beta-hydroxybutyrate dehydrogenase mitochondrial GN=BDH1 | 44.98 | 1 | 1 |  |  |  |  |  |  |  |  |  |
| Q9UBS4 | DnaJ homolog subfamily B member 11 GN=DNAJB11 | 30.56 | 1 | 1 |  |  |  |  |  |  |  |  |  |
| P24534 | Elongation factor 1-beta GN=EEF1B2 | 61.05 | 1 | 1 |  |  |  |  |  |  |  |  |  |
| Q15375 | Ephrin type-A receptor 7 GN=EPHA7 | 33.74 | 1 | 1 |  |  |  |  |  |  |  |  |  |
| Q9HAV4 | Exportin-5 GN=XPO5 | 42.76 | 1 | 1 |  |  |  |  |  |  |  |  |  |
| P09382 | Galectin-1 GN=LGALS1 | 41.47 | 1 | 1 |  |  |  |  |  |  |  |  |  |
| P11413 | Glucose-6-phosphate 1-dehydrogenase GN=G6PD | 30.81 | 1 | 1 |  |  |  |  |  |  |  |  |  |
| Q9HAV0 | Guanine nucleotide-binding protein subunit beta-4  GN=GNB4 | 33.74 | 1 | 1 |  |  |  |  |  |  |  |  |  |
| Q92769 | Histone deacetylase 2 GN=HDAC2 | 29.9 | 1 | 1 |  |  |  |  |  |  |  |  |  |
| Q02539 | Histone H1.1 GN=H1-1 | 41.99 | 1 | 1 |  |  |  |  |  |  |  |  |  |
| P16403 | Histone H1.2 GN=H1-2 | 41.99 | 1 | 1 |  |  |  |  |  |  |  |  |  |
| P16402 | Histone H1.3 GN=H1-3 | 41.99 | 1 | 1 |  |  |  |  |  |  |  |  |  |
| P10412 | Histone H1.4 GN=H1-4 | 41.99 | 1 | 1 |  |  |  |  |  |  |  |  |  |
| P22492 | Histone H1t GN=HIST1H1T | 41.99 | 1 | 1 |  |  |  |  |  |  |  |  |  |
| O00629 | Importin subunit alpha-3 GN=KPNA4 | 29.5 | 1 | 1 |  |  |  |  |  |  |  |  |  |
| Q71RC2 | La-related protein 4 GN=LARP4 | 29.46 | 1 | 1 |  |  |  |  |  |  |  |  |  |
| P24666 | Low molecular weight phosphotyrosine protein phosphatase GN=ACP1 | 37.62 | 1 | 1 |  |  |  |  |  |  |  |  |  |
| Q8WWC4 | m-AAA protease-interacting protein 1 mitochondrial  GN=MAIP1 | 32.99 | 1 | 1 |  |  |  |  |  |  |  |  |  |
| O43148 | mRNA cap guanine-N7 methyltransferase GN=RNMT | 29.96 | 1 | 1 |  |  |  |  |  |  |  |  |  |
| Q9HCE5 | N6-adenosine-methyltransferase non-catalytic subunit  GN=METTL14 | 28.81 | 1 | 1 |  |  |  |  |  |  |  |  |  |
| P43490 | Nicotinamide phosphoribosyltransferase GN=NAMPT | 37.09 | 1 | 1 |  |  |  |  |  |  |  |  |  |
| Q9NPP4 | NLR family CARD domain-containing protein 4  GN=NLRC4 | 28.92 | 1 | 1 |  |  |  |  |  |  |  |  |  |
| P19338 | Nucleolin GN=NCL | 34.31 | 1 | 1 |  |  |  |  |  |  |  |  |  |
| Q9UBD5 | Origin recognition complex subunit 3 GN=ORC3 | 33.42 | 1 | 1 |  |  |  |  |  |  |  |  |  |
| P62937 | Peptidyl-prolyl cis-trans isomerase A GN=PPIA | 36.96 | 1 | 1 |  |  |  |  |  |  |  |  |  |
| O00750 | Phosphatidylinositol 4-phosphate 3-kinase C2 domain containing subunit beta GN=PIK3C2B | 29.83 | 1 | 1 |  |  |  |  |  |  |  |  |  |
| P09874 | Poly [ADP-ribose] polymerase 1 GN=PARP1 | 68.85 | 1 | 1 |  |  |  |  |  |  |  |  |  |
| Q9NVV4 | Poly(A) RNA polymerase mitochondrial GN=MTPAP | 37.49 | 1 | 1 |  |  |  |  |  |  |  |  |  |
| Q8N490 | Probable hydrolase PNKD GN=PNKD | 47.36 | 1 | 1 |  |  |  |  |  |  |  |  |  |
| Q8WXW3 | Progesterone-induced-blocking factor 1 GN=PIBF1 | 35.12 | 1 | 1 |  |  |  |  |  |  |  |  |  |
| P11171 | Protein 4.1 GN=EPB41 | 28.64 | 1 | 1 |  |  |  |  |  |  |  |  |  |
| Q9BRX2 | Protein pelota homolog GN=PELO | 43.76 | 1 | 1 |  |  |  |  |  |  |  |  |  |
| P11177 | Pyruvate dehydrogenase E1 component subunit beta mitochondrial GN=PDHB | 39.55 | 1 | 1 |  |  |  |  |  |  |  |  |  |
| P28702 | Retinoic acid receptor RXR-beta GN=RXRB | 62.33 | 1 | 1 |  |  |  |  |  |  |  |  |  |
| Q9Y224 | RNA transcription translation and transport factor protein GN=RTRAF | 44.16 | 1 | 1 |  |  |  |  |  |  |  |  |  |
| A0AV96 | RNA-binding protein 47 GN=RBM47 | 48.45 | 1 | 1 |  |  |  |  |  |  |  |  |  |
| Q01844 | RNA-binding protein EWS GN=EWSR1 | 37.25 | 1 | 1 |  |  |  |  |  |  |  |  |  |
| O14983 | Sarcoplasmic/endoplasmic reticulum calcium ATPase 1 GN=ATP2A1 | 31.96 | 1 | 1 |  |  |  |  |  |  |  |  |  |
| P16615 | Sarcoplasmic/endoplasmic reticulum calcium ATPase 2 GN=ATP2A2 | 31.96 | 1 | 1 |  |  |  |  |  |  |  |  |  |
| P49458 | Signal recognition particle 9 kDa protein GN=SRP9 | 31.17 | 1 | 1 |  |  |  |  |  |  |  |  |  |
| Q8WVI0 | Small integral membrane protein 4 GN=SMIM4 | 44.13 | 1 | 1 |  |  |  |  |  |  |  |  |  |
| Q01081 | Splicing factor U2AF 35 kDa subunit GN=U2AF1 | 42.64 | 1 | 1 |  |  |  |  |  |  |  |  |  |
| P0DN76 | Splicing factor U2AF 35 kDa subunit-like protein  GN=U2AF1L5 | 42.64 | 1 | 1 |  |  |  |  |  |  |  |  |  |
| P31040 | Succinate dehydrogenase [ubiquinone] flavoprotein subunit mitochondrial GN=SDHA | 41.53 | 1 | 1 |  |  |  |  |  |  |  |  |  |
| Q16650 | T-box brain protein 1 GN=TBR1 | 29.39 | 1 | 1 |  |  |  |  |  |  |  |  |  |
| P17987 | T-complex protein 1 subunit alpha GN=TCP1 | 30.03 | 1 | 1 |  |  |  |  |  |  |  |  |  |
| Q9ULT0 | Tetratricopeptide repeat protein 7A GN=TTC7A | 37.64 | 1 | 1 |  |  |  |  |  |  |  |  |  |
| Q02086 | Transcription factor Sp2 GN=SP2 | 30.17 | 1 | 1 |  |  |  |  |  |  |  |  |  |
| Q13428 | Treacle protein GN=TCOF1 | 44.6 | 1 | 1 |  |  |  |  |  |  |  |  |  |
| P35030 | Trypsin-3 GN=PRSS3 | 32.89 | 1 | 1 |  |  |  |  |  |  |  |  |  |
| P49815 | Tuberin GN=TSC2 | 37.35 | 1 | 1 |  |  |  |  |  |  |  |  |  |
| E9PRG8 | Uncharacterized protein C11orf98 GN=C11orf98 | 40.4 | 1 | 1 |  |  |  |  |  |  |  |  |  |
| Q9NW07 | Zinc finger protein 358 GN=ZNF358 | 38.93 | 1 | 1 |  |  |  |  |  |  |  |  |  |
| Q8NDX6 | Zinc finger protein 740 GN=ZNF740 | 52.18 | 1 | 1 |  |  |  |  |  |  |  |  |  |
| P34931 | Heat shock 70 kDa protein 1-like GN=HSPA1L | 125.13 | 5 | 0 |  |  |  |  |  |  |  |  |  |
| P17066 | Heat shock 70 kDa protein 6 GN=HSPA6 | 97.49 | 4 | 0 |  |  |  |  |  |  |  |  |  |
| Q13885 | Tubulin beta-2A chain GN=TUBB2A | 203.54 | 9 | 0 |  |  |  |  |  |  |  |  |  |
| Q9BVA1 | Tubulin beta-2B chain GN=TUBB2B | 203.54 | 9 | 0 |  |  |  |  |  |  |  |  |  |
| Q13509 | Tubulin beta-3 chain GN=TUBB3 | 178.31 | 6 | 0 |  |  |  |  |  |  |  |  |  |
| Q9NZN5 | Rho guanine nucleotide exchange factor 12  GN=ARHGEF12 |  |  |  | 246.12 | 17 | 17 |  |  |  |  |  |  |
| Q5JTH9 | RRP12-like protein GN=RRP12 |  |  |  | 138.68 | 8 | 8 |  |  |  |  |  |  |
| Q96AG4 | Leucine-rich repeat-containing protein 59 GN=LRRC59 |  |  |  | 143 | 7 | 7 |  |  |  |  |  |  |
| P17544 | Cyclic AMP-dependent transcription factor ATF-7  GN=ATF7 |  |  |  | 133.36 | 7 | 7 |  |  |  |  |  |  |
| Q14974 | Importin subunit beta-1 GN=KPNB1 |  |  |  | 114.73 | 6 | 6 |  |  |  |  |  |  |
| P61586 | Transforming protein RhoA GN=RHOA |  |  |  | 146.78 | 6 | 6 |  |  |  |  |  |  |
| Q6SPF0 | Atherin GN=SAMD1 |  |  |  | 127.47 | 5 | 5 |  |  |  |  |  |  |
| O94906 | Pre-mRNA-processing factor 6 GN=PRPF6 |  |  |  | 104.73 | 5 | 5 |  |  |  |  |  |  |
| Q96QD9 | UAP56-interacting factor GN=FYTTD1 |  |  |  | 116.97 | 5 | 5 |  |  |  |  |  |  |
| Q14690 | Protein RRP5 homolog GN=PDCD11 |  |  |  | 96.11 | 4 | 4 |  |  |  |  |  |  |
| O14980 | Exportin-1 GN=XPO1 |  |  |  | 81.55 | 4 | 4 |  |  |  |  |  |  |
| Q29RF7 | Sister chromatid cohesion protein PDS5 homolog A GN=PDS5A |  |  |  | 109.4 | 4 | 4 |  |  |  |  |  |  |
| Q6P2Q9 | Pre-mRNA-processing-splicing factor 8 GN=PRPF8 |  |  |  | 95.74 | 4 | 4 |  |  |  |  |  |  |
| Q9H0S4 | Probable ATP-dependent RNA helicase DDX47  GN=DDX47 |  |  |  | 94.64 | 3 | 3 |  |  |  |  |  |  |
| Q5JTV8 | Torsin-1A-interacting protein 1 GN=TOR1AIP1 |  |  |  | 86.66 | 3 | 3 |  |  |  |  |  |  |
| Q14980 | Nuclear mitotic apparatus protein 1 GN=NUMA1 |  |  |  | 59.55 | 3 | 3 |  |  |  |  |  |  |
| Q96SI9 | Spermatid perinuclear RNA-binding protein GN=STRBP |  |  |  | 88.36 | 3 | 3 |  |  |  |  |  |  |
| Q9BUQ8 | Probable ATP-dependent RNA helicase DDX23  GN=DDX23 |  |  |  | 88.41 | 3 | 3 |  |  |  |  |  |  |
| P63000 | Ras-related C3 botulinum toxin substrate 1 GN=RAC1 |  |  |  | 89.08 | 3 | 3 |  |  |  |  |  |  |
| Q14684 | Ribosomal RNA processing protein 1 homolog B  GN=RRP1B |  |  |  | 76.59 | 3 | 3 |  |  |  |  |  |  |
| P04843 | Dolichyl-diphospho oligosaccharide protein glycosyltransferase subunit 1 GN=RPN1 |  |  |  | 90.32 | 3 | 3 |  |  |  |  |  |  |
| Q08209 | Serine/threonine-protein phosphatase 2B catalytic subunit alpha isoform GN=PPP3CA |  |  |  | 93.96 | 3 | 3 |  |  |  |  |  |  |
| Q9UNQ2 | Probable dimethyladenosine transferase GN=DIMT1 |  |  |  | 64.95 | 3 | 3 |  |  |  |  |  |  |
| P78316 | Nucleolar protein 14 GN=NOP14 |  |  |  | 76 | 3 | 3 |  |  |  |  |  |  |
| Q9NVP1 | ATP-dependent RNA helicase DDX18 GN=DDX18 |  |  |  | 68.59 | 3 | 3 |  |  |  |  |  |  |
| Q12788 | Transducin beta-like protein 3 GN=TBL3 |  |  |  | 109.36 | 3 | 3 |  |  |  |  |  |  |
| Q06587 | E3 ubiquitin-protein ligase RING1 GN=RING1 |  |  |  | 206.93 | 5 | 3 |  |  |  |  |  |  |
| P55317 | Hepatocyte nuclear factor 3-alpha GN=FOXA1 |  |  |  | 102.69 | 3 | 3 |  |  |  |  |  |  |
| Q8NI36 | WD repeat-containing protein 36 GN=WDR36 |  |  |  | 82.71 | 3 | 3 |  |  |  |  |  |  |
| P37268 | Squalene synthase GN=FDFT1 |  |  |  | 125.59 | 2 | 2 |  |  |  |  |  |  |
| P53621 | Coatomer subunit alpha GN=COPA |  |  |  | 61.68 | 2 | 2 |  |  |  |  |  |  |
| Q9UH99 | SUN domain-containing protein 2 GN=SUN2 |  |  |  | 67.79 | 2 | 2 |  |  |  |  |  |  |
| Q9H6R4 | Nucleolar protein 6 GN=NOL6 |  |  |  | 89.65 | 2 | 2 |  |  |  |  |  |  |
| Q9Y2U8 | Inner nuclear membrane protein Man1 GN=LEMD3 |  |  |  | 85.63 | 2 | 2 |  |  |  |  |  |  |
| P40938 | Replication factor C subunit 3 GN=RFC3 |  |  |  | 78.1 | 2 | 2 |  |  |  |  |  |  |
| P60468 | Protein transport protein Sec61 subunit beta  GN=SEC61B |  |  |  | 55.28 | 2 | 2 |  |  |  |  |  |  |
| P36776 | Lon protease homolog mitochondrial GN=LONP1 |  |  |  | 57.42 | 2 | 2 |  |  |  |  |  |  |
| Q8NI60 | Atypical kinase COQ8A mitochondrial GN=COQ8A |  |  |  | 45.78 | 2 | 2 |  |  |  |  |  |  |
| Q9HC52 | Chromobox protein homolog 8 GN=CBX8 |  |  |  | 90.67 | 2 | 2 |  |  |  |  |  |  |
| Q1ED39 | Lysine-rich nucleolar protein 1 GN=KNOP1 |  |  |  | 65.6 | 2 | 2 |  |  |  |  |  |  |
| Q99417 | c-Myc-binding protein GN=MYCBP |  |  |  | 69.26 | 2 | 2 |  |  |  |  |  |  |
| P46781 | 40S ribosomal protein S9 GN=RPS9 |  |  |  | 46.73 | 2 | 2 |  |  |  |  |  |  |
| P22415 | Upstream stimulatory factor 1 GN=USF1 |  |  |  | 67.51 | 2 | 2 |  |  |  |  |  |  |
| P40227 | T-complex protein 1 subunit zeta GN=CCT6A |  |  |  | 61.44 | 2 | 2 |  |  |  |  |  |  |
| Q14119 | Vascular endothelial zinc finger 1 GN=VEZF1 |  |  |  | 60.68 | 2 | 2 |  |  |  |  |  |  |
| Q9UMY1 | Nucleolar protein 7 GN=NOL7 |  |  |  | 59.8 | 2 | 2 |  |  |  |  |  |  |
| P0DOX5 | Immunoglobulin gamma-1 heavy chain |  |  |  | 30.26 | 1 | 1 |  |  |  |  |  |  |
| Q86VP6 | Cullin-associated NEDD8-dissociated protein 1 GN=CAND1 |  |  |  | 51.97 | 1 | 1 |  |  |  |  |  |  |
| Q86W42 | THO complex subunit 6 homolog GN=THOC6 |  |  |  | 29.41 | 1 | 1 |  |  |  |  |  |  |
| P20929 | Nebulin GN=NEB |  |  |  | 28.65 | 1 | 1 |  |  |  |  |  |  |
| Q9Y3A4 | Ribosomal RNA-processing protein 7 homolog A  GN=RRP7A |  |  |  | 37.95 | 1 | 1 |  |  |  |  |  |  |
| P0C0S5 | Histone H2A.Z GN=H2AZ1 |  |  |  | 35 | 1 | 1 |  |  |  |  |  |  |
| Q9BTM1 | Histone H2A.J GN=H2AFJ |  |  |  | 33.74 | 1 | 1 |  |  |  |  |  |  |
| P0C0S8 | Histone H2A type 1 GN=H2AC11 |  |  |  | 33.74 | 1 | 1 |  |  |  |  |  |  |
| O75533 | Splicing factor 3B subunit 1 GN=SF3B1 |  |  |  | 29.51 | 1 | 1 |  |  |  |  |  |  |
| Q7L7L0 | Histone H2A type 3 GN=HIST3H2A |  |  |  | 33.74 | 1 | 1 |  |  |  |  |  |  |
| Q8WY22 | BRI3-binding protein GN=BRI3BP |  |  |  | 57.94 | 1 | 1 |  |  |  |  |  |  |
| P51148 | Ras-related protein Rab-5C GN=RAB5C |  |  |  | 33.34 | 1 | 1 |  |  |  |  |  |  |
| P51587 | Breast cancer type 2 susceptibility protein GN=BRCA2 |  |  |  | 35.09 | 1 | 1 |  |  |  |  |  |  |
| P53618 | Coatomer subunit beta GN=COPB1 |  |  |  | 33.29 | 1 | 1 |  |  |  |  |  |  |
| P54886 | Delta-1-pyrroline-5-carboxylate synthase GN=ALDH18A1 |  |  |  | 43.81 | 1 | 1 |  |  |  |  |  |  |
| Q9Y5Q9 | General transcription factor 3C polypeptide 3  GN=GTF3C3 |  |  |  | 57.32 | 1 | 1 |  |  |  |  |  |  |
| O14556 | Glyceraldehyde-3-phosphate dehydrogenase testis specific GN=GAPDHS |  |  |  | 42.9 | 1 | 1 |  |  |  |  |  |  |
| Q13268 | Dehydrogenase/reductase SDR family member 2 mitochondrial GN=DHRS2 |  |  |  | 51.85 | 1 | 1 |  |  |  |  |  |  |
| Q99878 | Histone H2A type 1-J GN=H2AC14 |  |  |  | 33.74 | 1 | 1 |  |  |  |  |  |  |
| Q12931 | Heat shock protein 75 kDa mitochondrial GN=TRAP1 |  |  |  | 29.99 | 1 | 1 |  |  |  |  |  |  |
| Q8IYT2 | Cap-specific mRNA (nucleoside-2'-O-)-methyltransferase  2 GN=CMTR2 |  |  |  | 31.26 | 1 | 1 |  |  |  |  |  |  |
| O43615 | Mitochondrial import inner membrane translocase subunit  TIM44 GN=TIMM44 |  |  |  | 48.08 | 1 | 1 |  |  |  |  |  |  |
| P62826 | GTP-binding nuclear protein Ran GN=RAN |  |  |  | 44.74 | 1 | 1 |  |  |  |  |  |  |
| Q9NVH2 | Integrator complex subunit 7 GN=INTS7 |  |  |  | 31.09 | 1 | 1 |  |  |  |  |  |  |
| P62979 | Ubiquitin-40S ribosomal protein S27a GN=RPS27A |  |  |  | 33.65 | 1 | 1 |  |  |  |  |  |  |
| Q86X55 | Histone-arginine methyltransferase CARM1 GN=CARM1 |  |  |  | 37.36 | 1 | 1 |  |  |  |  |  |  |
| Q96DT7 | Zinc finger and BTB domain-containing protein 10  GN=ZBTB10 |  |  |  | 63.41 | 1 | 1 |  |  |  |  |  |  |
| O96019 | Actin-like protein 6A GN=ACTL6A |  |  |  | 31.01 | 1 | 1 |  |  |  |  |  |  |
| P61803 | Dolichyl-diphospho oligosaccharide protein glycosyltransferase subunit DAD1 GN=DAD1 |  |  |  | 51.83 | 1 | 1 |  |  |  |  |  |  |
| Q8NAF0 | Zinc finger protein 579 GN=ZNF579 |  |  |  | 46.57 | 1 | 1 |  |  |  |  |  |  |
| P84077 | ADP-ribosylation factor 1 GN=ARF1 |  |  |  | 55.09 | 1 | 1 |  |  |  |  |  |  |
| Q99988 | Growth/differentiation factor 15 GN=GDF15 |  |  |  | 55.04 | 1 | 1 |  |  |  |  |  |  |
| Q9NRL2 | Bromodomain adjacent to zinc finger domain protein 1A  GN=BAZ1A |  |  |  | 48.09 | 1 | 1 |  |  |  |  |  |  |
| Q8N3C7 | CAP-Gly domain-containing linker protein 4 GN=CLIP4 |  |  |  | 37.61 | 1 | 1 |  |  |  |  |  |  |
| P62820 | Ras-related protein Rab-1A GN=RAB1A |  |  |  | 67.75 | 2 | 1 |  |  |  |  |  |  |
| P20671 | Histone H2A type 1-D GN=H2AC7 |  |  |  | 33.74 | 1 | 1 |  |  |  |  |  |  |
| P62987 | Ubiquitin-60S ribosomal protein L40 GN=UBA52 |  |  |  | 33.65 | 1 | 1 |  |  |  |  |  |  |
| P84085 | ADP-ribosylation factor 5 GN=ARF5 |  |  |  | 55.09 | 1 | 1 |  |  |  |  |  |  |
| Q76N89 | E3 ubiquitin-protein ligase HECW1 GN=HECW1 |  |  |  | 28.94 | 1 | 1 |  |  |  |  |  |  |
| P0CG48 | Polyubiquitin-C GN=UBC |  |  |  | 33.65 | 1 | 1 |  |  |  |  |  |  |
| Q9NYU1 | UDP-glucose glycoprotein glucosyltransferase 2  GN=UGGT2 |  |  |  | 29.8 | 1 | 1 |  |  |  |  |  |  |
| Q9BZF1 | Oxysterol-binding protein-related protein 8 GN=OSBPL8 |  |  |  | 44.69 | 1 | 1 |  |  |  |  |  |  |
| P20226 | TATA-box-binding protein GN=TBP |  |  |  | 64.95 | 1 | 1 |  |  |  |  |  |  |
| Q49A26 | Putative oxidoreductase GLYR1 GN=GLYR1 |  |  |  | 31.78 | 1 | 1 |  |  |  |  |  |  |
| P20339 | Ras-related protein Rab-5A GN=RAB5A |  |  |  | 33.34 | 1 | 1 |  |  |  |  |  |  |
| Q9H0U4 | Ras-related protein Rab-1B GN=RAB1B |  |  |  | 67.75 | 2 | 1 |  |  |  |  |  |  |
| Q16777 | Histone H2A type 2-C GN=HIST2H2AC |  |  |  | 33.74 | 1 | 1 |  |  |  |  |  |  |
| Q16718 | NADH dehydrogenase [ubiquinone] 1 alpha subcomplex subunit 5 GN=NDUFA5 |  |  |  | 32.19 | 1 | 1 |  |  |  |  |  |  |
| P61204 | ADP-ribosylation factor 3 GN=ARF3 |  |  |  | 55.09 | 1 | 1 |  |  |  |  |  |  |
| Q5SRE5 | Nucleoporin NUP188 homolog GN=NUP188 |  |  |  | 32.63 | 1 | 1 |  |  |  |  |  |  |
| P35251 | Replication factor C subunit 1 GN=RFC1 |  |  |  | 46.74 | 1 | 1 |  |  |  |  |  |  |
| Q8TF72 | Protein Shroom3 GN=SHROOM3 |  |  |  | 28.27 | 1 | 1 |  |  |  |  |  |  |
| Q86WA8 | Lon protease homolog 2 peroxisomal GN=LONP2 |  |  |  | 34.67 | 1 | 1 |  |  |  |  |  |  |
| Q8N1F7 | Nuclear pore complex protein Nup93 GN=NUP93 |  |  |  | 36.96 | 1 | 1 |  |  |  |  |  |  |
| P0CG47 | Polyubiquitin-B GN=UBB |  |  |  | 33.65 | 1 | 1 |  |  |  |  |  |  |
| Q9H0M5 | Zinc finger protein 700 GN=ZNF700 |  |  |  | 31.24 | 1 | 1 |  |  |  |  |  |  |
| P01860 | Immunoglobulin heavy constant gamma 3 GN=IGHG3 |  |  |  | 30.26 | 1 | 1 |  |  |  |  |  |  |
| Q8N1G0 | Zinc finger protein 687 GN=ZNF687 |  |  |  | 30.26 | 1 | 1 |  |  |  |  |  |  |
| P50991 | T-complex protein 1 subunit delta GN=CCT4 |  |  |  | 69.03 | 1 | 1 |  |  |  |  |  |  |
| A6NKH3 | Putative 60S ribosomal protein L37a-like protein  GN=RPL37AP8 |  |  |  | 28.48 | 1 | 1 |  |  |  |  |  |  |
| P62316 | Small nuclear ribonucleoprotein Sm D2 GN=SNRPD2 |  |  |  | 31.81 | 1 | 1 |  |  |  |  |  |  |
| P61020 | Ras-related protein Rab-5B GN=RAB5B |  |  |  | 33.34 | 1 | 1 |  |  |  |  |  |  |
| P35249 | Replication factor C subunit 4 GN=RFC4 |  |  |  | 59.14 | 1 | 1 |  |  |  |  |  |  |
| Q15269 | Periodic tryptophan protein 2 homolog GN=PWP2 |  |  |  | 42.48 | 1 | 1 |  |  |  |  |  |  |
| Q93077 | Histone H2A type 1-C GN=HIST1H2AC |  |  |  | 33.74 | 1 | 1 |  |  |  |  |  |  |
| Q92928 | Putative Ras-related protein Rab-1C GN=RAB1C |  |  |  | 67.75 | 2 | 1 |  |  |  |  |  |  |
| Q71UI9 | Histone H2A.V GN=H2AFV |  |  |  | 35 | 1 | 1 |  |  |  |  |  |  |
| P61026 | Ras-related protein Rab-10 GN=RAB10 |  |  |  | 60.96 | 2 | 1 |  |  |  |  |  |  |
| Q13823 | Nucleolar GTP-binding protein 2 GN=GNL2 |  |  |  | 57.67 | 1 | 1 |  |  |  |  |  |  |
| Q9BVP2 | Guanine nucleotide-binding protein-like 3 GN=GNL3 |  |  |  | 38.6 | 1 | 1 |  |  |  |  |  |  |
| Q13185 | Chromobox protein homolog 3 GN=CBX3 |  |  |  | 54.15 | 1 | 1 |  |  |  |  |  |  |
| Q92552 | 28S ribosomal protein S27 mitochondrial GN=MRPS27 |  |  |  | 32.79 | 1 | 1 |  |  |  |  |  |  |
| Q9Y383 | Putative RNA-binding protein Luc7-like 2 GN=LUC7L2 |  |  |  | 52.96 | 1 | 1 |  |  |  |  |  |  |
| P15954 | Cytochrome c oxidase subunit 7C mitochondrial  GN=COX7C |  |  |  | 37.67 | 1 | 1 |  |  |  |  |  |  |
| Q01344 | Interleukin-5 receptor subunit alpha GN=IL5RA |  |  |  | 28.96 | 1 | 1 |  |  |  |  |  |  |
| P01857 | Immunoglobulin heavy constant gamma 1 GN=IGHG1 |  |  |  | 30.26 | 1 | 1 |  |  |  |  |  |  |
| Q14692 | Ribosome biogenesis protein BMS1 homolog GN=BMS1 |  |  |  | 29.2 | 1 | 1 |  |  |  |  |  |  |
| P49792 | E3 SUMO-protein ligase RanBP2 GN=RANBP2 |  |  |  | 28.36 | 1 | 1 |  |  |  |  |  |  |
| Q03701 | CCAAT/enhancer-binding protein zeta GN=CEBPZ |  |  |  | 34.2 | 1 | 1 |  |  |  |  |  |  |
| Q9NZM5 | Ribosome biogenesis protein NOP53 GN=NOP53 |  |  |  | 67.59 | 1 | 1 |  |  |  |  |  |  |
| Q9Y5M8 | Signal recognition particle receptor subunit beta  GN=SRPRB |  |  |  | 39.13 | 1 | 1 |  |  |  |  |  |  |
| Q8N8A6 | ATP-dependent RNA helicase DDX51 GN=DDX51 |  |  |  | 54.22 | 1 | 1 |  |  |  |  |  |  |
| Q96EL3 | 39S ribosomal protein L53 mitochondrial GN=MRPL53 |  |  |  | 48.43 | 1 | 1 |  |  |  |  |  |  |
| Q8N183 | NADH dehydrogenase [ubiquinone] 1 alpha subcomplex assembly factor 2 GN=NDUFAF2 |  |  |  | 29.27 | 1 | 1 |  |  |  |  |  |  |
| Q5CZC0 | Fibrous sheath-interacting protein 2 GN=FSIP2 |  |  |  | 30.4 | 1 | 1 |  |  |  |  |  |  |
| Q7Z7H8 | 39S ribosomal protein L10 mitochondrial GN=MRPL10 |  |  |  | 31.17 | 1 | 1 |  |  |  |  |  |  |
| Q96KK5 | Histone H2A type 1-H GN=HIST1H2AH |  |  |  | 33.74 | 1 | 1 |  |  |  |  |  |  |
| Q9H0U9 | Testis-specific Y-encoded-like protein 1 GN=TSPYL1 |  |  |  | 31.04 | 1 | 1 |  |  |  |  |  |  |
| O43929 | Origin recognition complex subunit 4 GN=ORC4 |  |  |  | 30.81 | 1 | 1 |  |  |  |  |  |  |
| A8MTT3 | Protein CEBPZOS GN=CEBPZOS |  |  |  | 37.83 | 1 | 1 |  |  |  |  |  |  |
| O43913 | Origin recognition complex subunit 5 GN=ORC5 |  |  |  | 39.75 | 1 | 1 |  |  |  |  |  |  |
| Q6FI13 | Histone H2A type 2-A GN=HIST2H2AA3 |  |  |  | 33.74 | 1 | 1 |  |  |  |  |  |  |
| P04908 | Histone H2A type 1-B/E GN=H2AC4 |  |  |  | 33.74 | 1 | 1 |  |  |  |  |  |  |
| Q14669 | E3 ubiquitin-protein ligase TRIP12 GN=TRIP12 |  |  |  | 45.78 | 1 | 1 |  |  |  |  |  |  |
| P42166 | Lamina-associated polypeptide 2 isoform alpha  GN=TMPO |  |  |  | 172.68 | 7 | 1 |  |  |  |  |  |  |
| Q9C005 | Protein dpy-30 homolog GN=DPY30 |  |  |  | 67.66 | 1 | 1 |  |  |  |  |  |  |
| Q8TED0 | U3 small nucleolar RNA-associated protein 15 homolog GN=UTP15 |  |  |  | 45.15 | 1 | 1 |  |  |  |  |  |  |
| Q92797 | Symplekin GN=SYMPK |  |  |  | 34.57 | 1 | 1 |  |  |  |  |  |  |
| Q6DKI1 | 60S ribosomal protein L7-like 1 GN=RPL7L1 |  |  |  | 35.96 | 1 | 1 |  |  |  |  |  |  |
| Q14244 | Ensconsin GN=MAP7 |  |  |  | 28.69 | 1 | 1 |  |  |  |  |  |  |
| Q96DB2 | Histone deacetylase 11 GN=HDAC11 |  |  |  | 30.52 | 1 | 1 |  |  |  |  |  |  |
| Q9UNX3 | 60S ribosomal protein L26-like 1 GN=RPL26L1 |  |  |  | 29.13 | 1 | 1 |  |  |  |  |  |  |
| Q93009 | Ubiquitin carboxyl-terminal hydrolase 7 GN=USP7 |  |  |  | 67.26 | 1 | 1 |  |  |  |  |  |  |
| Q92621 | Nuclear pore complex protein Nup205 GN=NUP205 |  |  |  | 32.98 | 1 | 1 |  |  |  |  |  |  |
| P49368 | T-complex protein 1 subunit gamma GN=CCT3 |  |  |  | 71.76 | 1 | 1 |  |  |  |  |  |  |
| P07900 | Heat shock protein HSP 90-alpha GN=HSP90AA1 |  |  |  | 29.99 | 1 | 1 |  |  |  |  |  |  |
| P57721 | Poly(rC)-binding protein 3 GN=PCBP3 |  |  |  |  |  |  | 32.97 | 1 | 0 |  |  |  |
| P39023 | 60S ribosomal protein L3 GN=RPL3 |  |  |  |  |  |  | 46.84 | 1 | 1 |  |  |  |
| Q14525 | Keratin type I cuticular Ha3-II GN=KRT33B |  |  |  |  |  |  | 35.74 | 1 | 1 |  |  |  |
| Q16740 | ATP-dependent Clp protease proteolytic subunit mitochondrial GN=CLPP |  |  |  |  |  |  | 33.49 | 1 | 1 |  |  |  |
| Q02447 | Transcription factor Sp3 GN=SP3 |  |  |  |  |  |  | 33.05 | 1 | 1 |  |  |  |
| P14923 | Junction plakoglobin GN=JUP |  |  |  |  |  |  | 31.78 | 1 | 1 |  |  |  |
| Q9BQ39 | ATP-dependent RNA helicase DDX50 GN=DDX50 |  |  |  |  |  |  | 46.85 | 1 | 1 |  |  |  |
| Q2M1P5 | Kinesin-like protein KIF7 GN=KIF7 |  |  |  |  |  |  | 34.63 | 1 | 1 |  |  |  |
| P20674 | Cytochrome c oxidase subunit 5A mitochondrial  GN=COX5A |  |  |  |  |  |  | 40.36 | 1 | 1 |  |  |  |
| Q8IX03 | Protein KIBRA GN=WWC1 |  |  |  |  |  |  | 31.14 | 1 | 1 |  |  |  |
| P12235 | ADP/ATP translocase 1 GN=SLC25A4 |  |  |  |  |  |  | 48.96 | 1 | 1 |  |  |  |
| Q3ZCT1 | Zinc finger protein 260 GN=ZNF260 |  |  |  |  |  |  | 52.02 | 1 | 1 |  |  |  |
| Q96FJ2 | Dynein light chain 2 cytoplasmic GN=DYNLL2 |  |  |  |  |  |  | 31.44 | 1 | 1 |  |  |  |
| P81605 | Dermcidin GN=DCD |  |  |  |  |  |  | 47.19 | 1 | 1 |  |  |  |
| Q00536 | Cyclin-dependent kinase 16 GN=CDK16 |  |  |  |  |  |  | 34.25 | 1 | 1 |  |  |  |
| Q9NQ39 | Putative 40S ribosomal protein S10-like GN=RPS10P5 |  |  |  |  |  |  | 72.29 | 2 | 2 |  |  |  |
| P31930 | Cytochrome b-c1 complex subunit 1 mitochondrial  GN=UQCRC1 |  |  |  |  |  |  |  |  |  | 105.29 | 3 | 3 |
| Q9BSJ8 | Extended synaptotagmin-1 GN=ESYT1 |  |  |  |  |  |  |  |  |  | 62.63 | 2 | 2 |
| P21912 | Succinate dehydrogenase [ubiquinone] iron-sulphur subunit mitochondrial GN=SDHB |  |  |  |  |  |  |  |  |  | 68.28 | 2 | 2 |
| Q8N3Y7 | Epidermal retinol dehydrogenase 2 GN=SDR16C5 |  |  |  |  |  |  |  |  |  | 76.02 | 2 | 2 |
| P14625 | Endoplasmin GN=HSP90B1 |  |  |  |  |  |  |  |  |  | 72.67 | 2 | 2 |
| P02656 | Apolipoprotein C-III GN=APOC3 |  |  |  |  |  |  |  |  |  | 53.75 | 1 | 1 |
| Q9Y3D7 | Mitochondrial import inner membrane translocase subunit TIM16 GN=PAM16 |  |  |  |  |  |  |  |  |  | 48.96 | 1 | 1 |
| P15336 | Cyclic AMP-dependent transcription factor ATF-2  GN=ATF2 |  |  |  |  |  |  |  |  |  | 49.29 | 1 | 1 |
| P13073 | Cytochrome c oxidase subunit 4 isoform 1 mitochondrial GN=COX4I1 |  |  |  |  |  |  |  |  |  | 38.83 | 1 | 1 |
| Q71U36 | Tubulin alpha-1A chain GN=TUBA1A |  |  |  |  |  |  |  |  |  | 45.07 | 1 | 1 |
| P35232 | Prohibitin GN=PHB |  |  |  |  |  |  |  |  |  | 36.41 | 1 | 1 |
| O14979 | Heterogeneous nuclear ribonucleoprotein D-like  GN=HNRNPDL |  |  |  |  |  |  |  |  |  | 41.47 | 1 | 1 |
| Q9NY65 | Tubulin alpha-8 chain GN=TUBA8 |  |  |  |  |  |  |  |  |  | 45.07 | 1 | 1 |
| P05023 | Sodium/potassium-transporting ATPase subunit alpha-1 GN=ATP1A1 |  |  |  |  |  |  |  |  |  | 40.14 | 1 | 1 |
| Q9UL25 | Ras-related protein Rab-21 GN=RAB21 |  |  |  |  |  |  |  |  |  | 50.83 | 1 | 1 |
| O95202 | Mitochondrial proton/calcium exchanger protein  GN=LETM1 |  |  |  |  |  |  |  |  |  | 47.82 | 1 | 1 |
| A6NIZ1 | Ras-related protein Rap-1b-like protein |  |  |  |  |  |  |  |  |  | 41.45 | 1 | 1 |
| P47985 | Cytochrome b-c1 complex subunit Rieske mitochondrial GN=UQCRFS1 |  |  |  |  |  |  |  |  |  | 38.18 | 1 | 1 |
| P68366 | Tubulin alpha-4A chain GN=TUBA4A |  |  |  |  |  |  |  |  |  | 45.07 | 1 | 1 |
| Q96DA6 | Mitochondrial import inner membrane translocase subunit TIM14 GN=DNAJC19 |  |  |  |  |  |  |  |  |  | 40.9 | 1 | 1 |
| Q15286 | Ras-related protein Rab-35 GN=RAB35 |  |  |  |  |  |  |  |  |  | 39.91 | 1 | 1 |
| P50993 | Sodium/potassium-transporting ATPase subunit alpha-2 GN=ATP1A2 |  |  |  |  |  |  |  |  |  | 40.14 | 1 | 1 |
| P0DPH8 | Tubulin alpha-3D chain GN=TUBA3D |  |  |  |  |  |  |  |  |  | 45.07 | 1 | 1 |
| O75915 | PRA1 family protein 3 GN=ARL6IP5 |  |  |  |  |  |  |  |  |  | 39.36 | 1 | 1 |
| Q6PEY2 | Tubulin alpha-3E chain GN=TUBA3E |  |  |  |  |  |  |  |  |  | 45.07 | 1 | 1 |
| Q04695 | Keratin type I cytoskeletal 17 GN=KRT17 |  |  |  |  |  |  |  |  |  | 53.25 | 1 | 1 |
| A6NHL2 | Tubulin alpha chain-like 3 GN=TUBAL3 |  |  |  |  |  |  |  |  |  | 45.07 | 1 | 1 |
| Q9UDW1 | Cytochrome b-c1 complex subunit 9 GN=UQCR10 |  |  |  |  |  |  |  |  |  | 35.64 | 1 | 1 |
| P61019 | Ras-related protein Rab-2A GN=RAB2A |  |  |  |  |  |  |  |  |  | 36.94 | 1 | 1 |
| Q9BYE2 | Transmembrane protease serine 13 GN=TMPRSS13 PE=2 SV=5 |  |  |  |  |  |  |  |  |  | 51.31 | 1 | 1 |
| P62834 | Ras-related protein Rap-1A GN=RAP1A |  |  |  |  |  |  |  |  |  | 41.45 | 1 | 1 |
| P29590 | Protein PML GN=PML |  |  |  |  |  |  |  |  |  | 42.71 | 1 | 1 |
| O00217 | NADH dehydrogenase [ubiquinone] iron-sulphur protein 8 mitochondrial GN=NDUFS8 |  |  |  |  |  |  |  |  |  | 37.61 | 1 | 1 |
| Q05639 | Elongation factor 1-alpha 2 GN=EEF1A2 |  |  |  |  |  |  |  |  |  | 38.43 | 1 | 1 |
| P0DPH7 | Tubulin alpha-3C chain GN=TUBA3C |  |  |  |  |  |  |  |  |  | 45.07 | 1 | 1 |
| Q02413 | Desmoglein-1 GN=DSG1 |  |  |  |  |  |  |  |  |  | 41.87 | 1 | 1 |
| P14927 | Cytochrome b-c1 complex subunit 7 GN=UQCRB |  |  |  |  |  |  |  |  |  | 51.81 | 1 | 1 |
| P13637 | Sodium/potassium-transporting ATPase subunit alpha-3  GN=ATP1A3 |  |  |  |  |  |  |  |  |  | 40.14 | 1 | 1 |
